# Addendum: Genomic analysis on pygmy hog reveals extensive interbreeding during wild boar expansion

**DOI:** 10.1038/s41467-020-20106-2

**Published:** 2020-12-03

**Authors:** Langqing Liu, Mirte Bosse, Hendrik-Jan Megens, Laurent A. F. Frantz, Young-Lim Lee, Evan K. Irving-Pease, Goutam Narayan, Martien A. M. Groenen, Ole Madsen

**Affiliations:** 1 Animal Breeding and Genomics, Wageningen University & Research, 6708PB Wageningen, the Netherlands; 2 School of Biological and Chemical Sciences, Queen Mary University of London, E1 4NS London, United Kingdom; 3 Palaeogenomics and Bioarcheology Research Network, Research Laboratory for Archeology and History of Art, University of Oxford, Oxford, OX1 3QY United Kingdom; 4 Durrell Wildlife Conservation Trust, Les Augrès Manor, Jersey, JE3 5BP Channel Islands United Kingdom; 5 Pygmy Hog Conservation Programme, EcoSystems-India, Indira Nagar, Basistha, Guwahati, Assam 781029 India

**Keywords:** Phylogenetics, Population genetics, Genetic hybridization, Genomics, Phylogenomics

Correction to: *Nature Communications* 10.1038/s41467-019-10017-2, published online 30 April 2019.

In the original article, we analyzed 38 genomes from pygmy hog and related suid species. Our analysis not only identified a signal of introgression between *Sus scrofa* and pygmy hog but also reinforced the idea that there was gene flow between *Sus scrofa* and an extinct (ghost) *Suidae* linage^1^. In our original analysis, however, for the *D*-statistics equation in Admixtools^2^, we mistakenly interpreted as $D = \frac{{{\rm{ABBA}} - {\rm{BABA}}}}{{{\rm{ABBA}} + {\rm{BABA}}}}$, while it was in fact $D = \frac{{{\rm{BABA}} - {\rm{ABBA}}}}{{{\rm{ABBA}} + {\rm{BABA}}}}$. This led to an inversion of the direction of admixture shown in Fig. 2b in the original version of the article. To remain consistent with the rest of the analyses in the article, we rectified the formula of *D*-statistics as $D = \frac{{{\rm{ABBA}} - {\rm{BABA}}}}{{{\rm{ABBA}} + {\rm{BABA}}}}$ and updated Fig. 2b (Fig. 1; this addendum). The results after correcting the formula indicate that in fact there is an excess of shared derived alleles between the pygmy hog and Island of Southeast Asian (ISEA) *Sus* (ABBA) and not between the pygmy hog and *Sus scrofa* (BABA). The excess of ABBA can be the result of admixture either between pygmy hog and ISEA *Sus* or between *Sus scrofa* and the archaic ghost linage or both. Methods to detect hybridization such as Patterson’s *D*, fd, or Twisst^3–5^, however, are inadequate to distinguish gene-flow events between P1 and P3 (Fig. 2, orange arrow) from those between P2 and an archaic ghost lineage basal to P3 (Fig. 2, blue arrow).

To address this issue an alternative method was used, based on relative nucleotide distance, as implemented in RND_min_^6^ to calculate the relative node depth between each taxon. To do so, we first used BEAGLE v4.1^7^ to perform haplotype phasing on genotypes of all individuals (excluding *Babyrousa babyrussa*, *Potomochoerus larvatus*, and *Potomochoerus porcus*) with default parameters. Then we calculated RND_min_ in 100-kb sliding windows along autosomes with <50% missing sites using PopGenome^8^. Comparisons were carried out between pygmy hog, ISEA *Sus*, and *Sus scrofa*, using the genome of a warthog as an outgroup. This shows that, for the genomic regions supporting topoA (Fig. 3a), the distribution of RND_min_ computed between the pygmy hog and *Sus scrofa* is significantly lower (16.2%, Welch’s *t* test *p* value <1.8e−14) than RND_min_ between pygmy hog and ISEA *Sus*, indicating that the first two species share more similarities. We also note that this pattern is more apparent in regions supporting topoA than in the overall genomic background (gray dots in Fig. 3a). Altogether, this indicates that the average genomic distance between the pygmy hog and *S. scrofa* is smaller than that between the pygmy hog and the ISEA *Sus* clade, which supports the existence of gene flow between pygmy hog and *S. scrofa*.

If the result of our *D*-statistics were affected by an admixture between pygmy hog and ISEA *Sus*, we would expect that, in regions that display topoB (Fig. 3b), the RND_min_ computed between the pygmy hog and ISEA *Sus* should be lower than in the overall genomic background. Our analysis, however, shows that this is not the case. In fact, we found these distributions to be very similar (Fig. 3b), especially when compared to the result shown in Fig. 3a. In addition, we found the distance between ISEA *Sus* and *Sus scrofa* to be higher (23.2%, Welch’s *t* test *p* value <2.2e−16) than the overall genomic background in the regions that show topoB, suggesting that topoB was caused by the admixture with an unsampled taxon.

Altogether, our analyses indicate that the findings and interpretations presented in the original article are correct. First, there was no admixture between ISEA *Sus* and pygmy hog. Instead, the combined results of the *D*-statistics and RND_min_ are more consistent with a ghost admixture between a distantly related taxon and *S. scrofa*, and this inflated the number of shared derived alleles between ISEA *Sus* and the pygmy hog. Second, the shorter distance between *Sus scrofa* and pygmy hog suggests that there was also admixture between these two species.

**Fig. 1:**
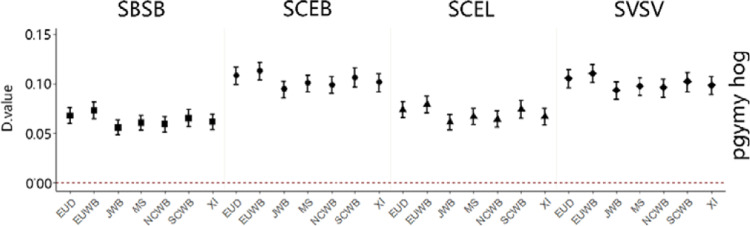
A diagram depicting the excess derived allele sharing when comparing sister taxa and outgroups. Each column contains the fraction of excess allele sharing by a taxon (up/down) with the pygmy hog/outgroup compared to its sister taxon (up/down). We computed *D* statistics of the form *D* (*X*, *Y*, Pygmy hog, warthog). Error bars correspond to three standard errors. (SBSB *Sus barbatus*, SCEB *Sus cebrifons*, SCEL *Sus celebensis*, SVSV *Sus verrucosus*, EUD European domesticated pig, EUWB European wild boar, JWB Japanese wild boar, MS Meishan, NCWB Northern China wild boar, SCWB Southern China wild boar, XI Xiang).

**Fig. 2:**
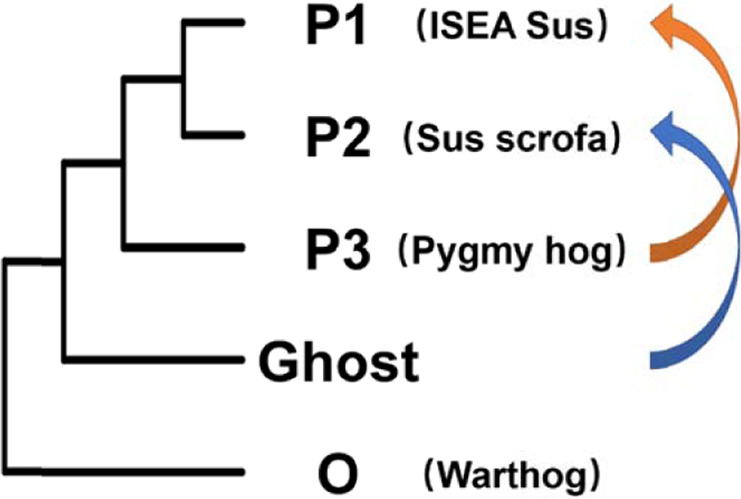
Schematic representation of the potential gene-flow scenarios discussed in the text. Two-way arrows pointing the two hybridized species.

**Fig. 3:**
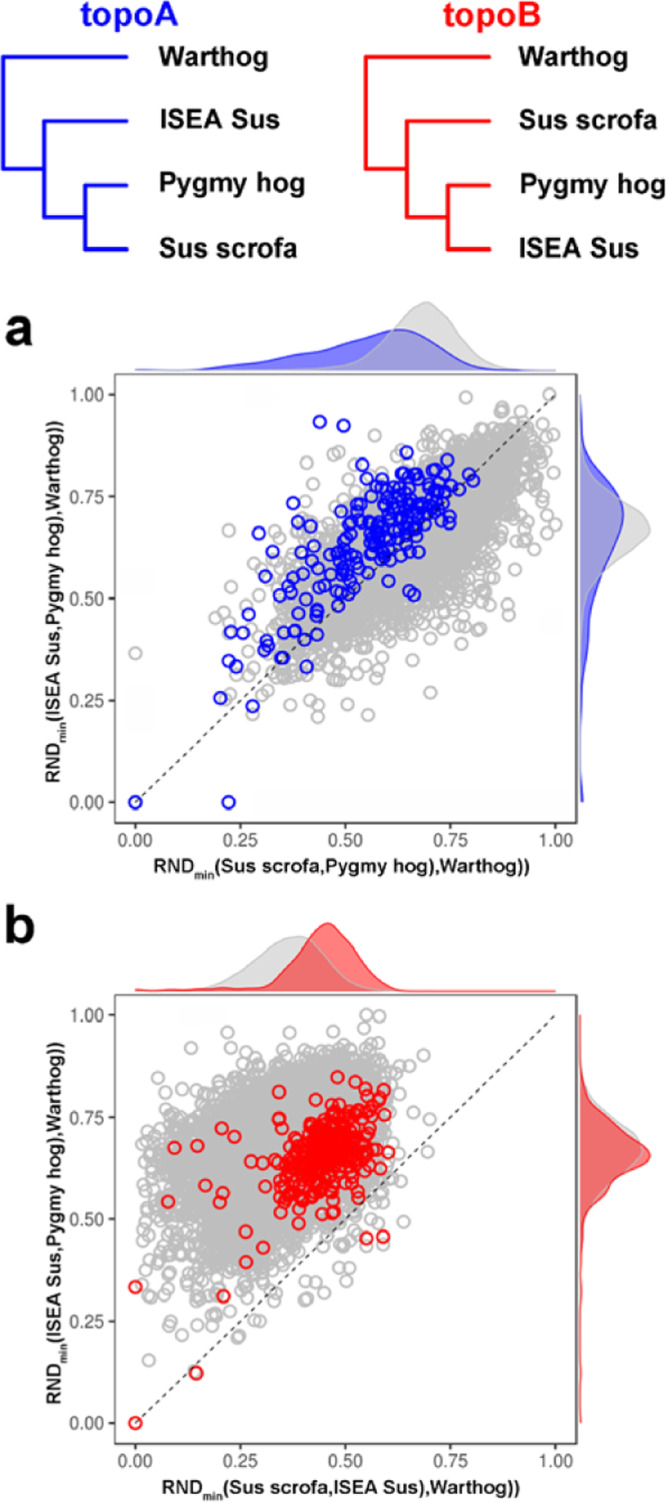
Phylogenetic tree indicated the alternative topologies (topoA and topoB) to the main species tree. Scatter and density plots below show the distribution of RND_min_ between different comparisons. **a** Blue circles represent RND_min_ distribution within windows supporting topoA. **b** Red circles represent RND_min_ distribution within windows supporting topoB. Gray circles represent RND_min_ distribution of the 100-kb windows among all autosomes. Populations used to calculate RND_min_ is shown on axis labels. Warthog was used as outgroup.
