# Supplementary material for: Genomic analysis on pygmy hog reveals extensive interbreeding during wild boar expansion
Source: Nat Commun. 2019 Apr 30;10:1992. doi: 10.1038/s41467-019-10017-2 (PMC6491599; doi:10.1038/s41467-019-10017-2)
Supplement: Supplementary file 1 — Supplementary Information [file 41467_2019_10017_MOESM1_ESM.pdf]

Supplementary Information

Genomic analysis on pygmy hog (*Porcula salvania*) reveals multiple interbreeding during wild boar expansion

Liu et al.

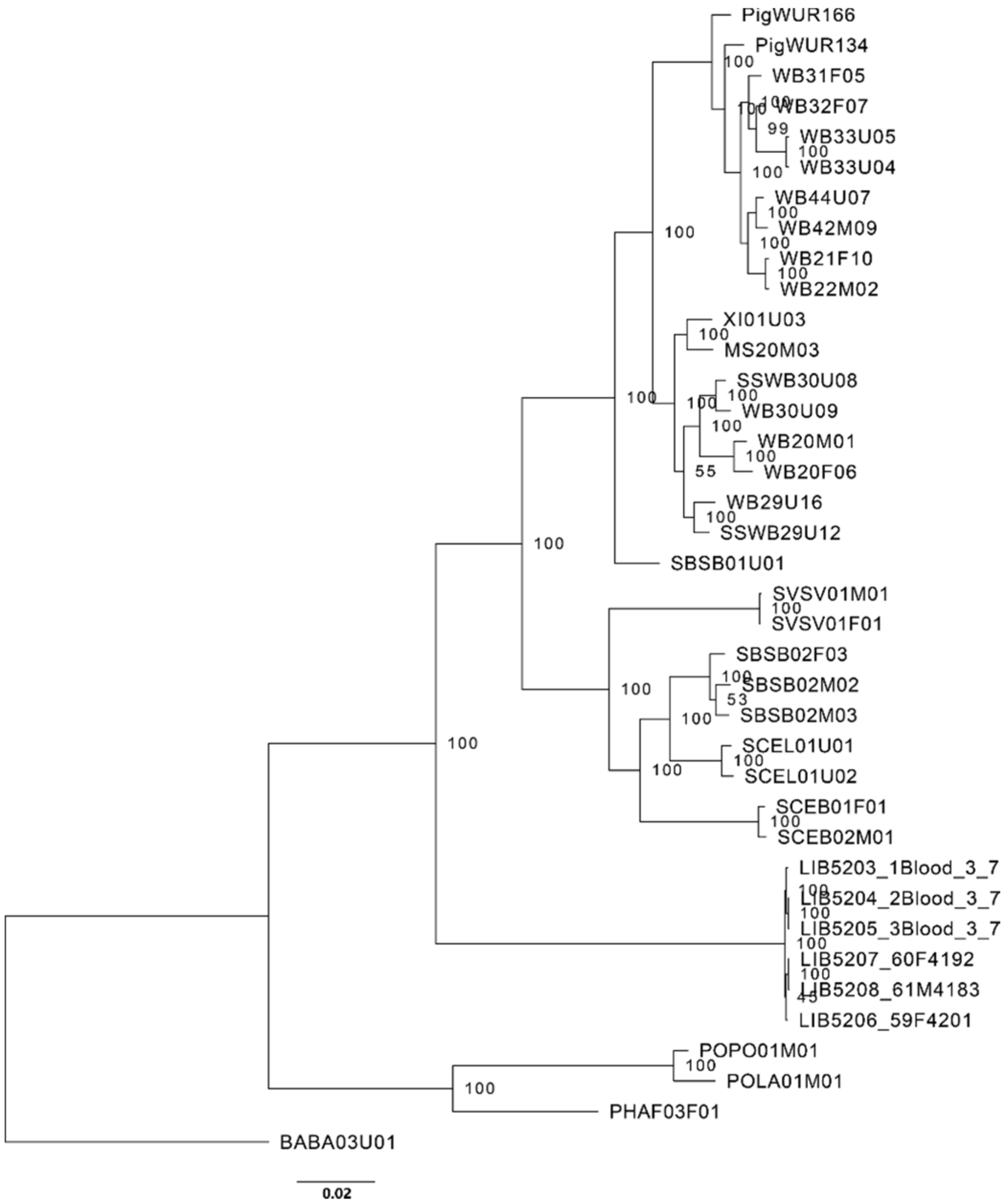

Supplementary Fig. 1

Maximum likelihood phylogenetic tree constructed on concatenated coding sequences using the supermatrix method (Optimization Likelihood: -42825135.3). The numbers on the nodes represent bootstrap values based on 100 non-parametric bootstrap replications. *Babyrousa babyrussa* was used as out group. See Supplementary data 1 for name abbreviation.

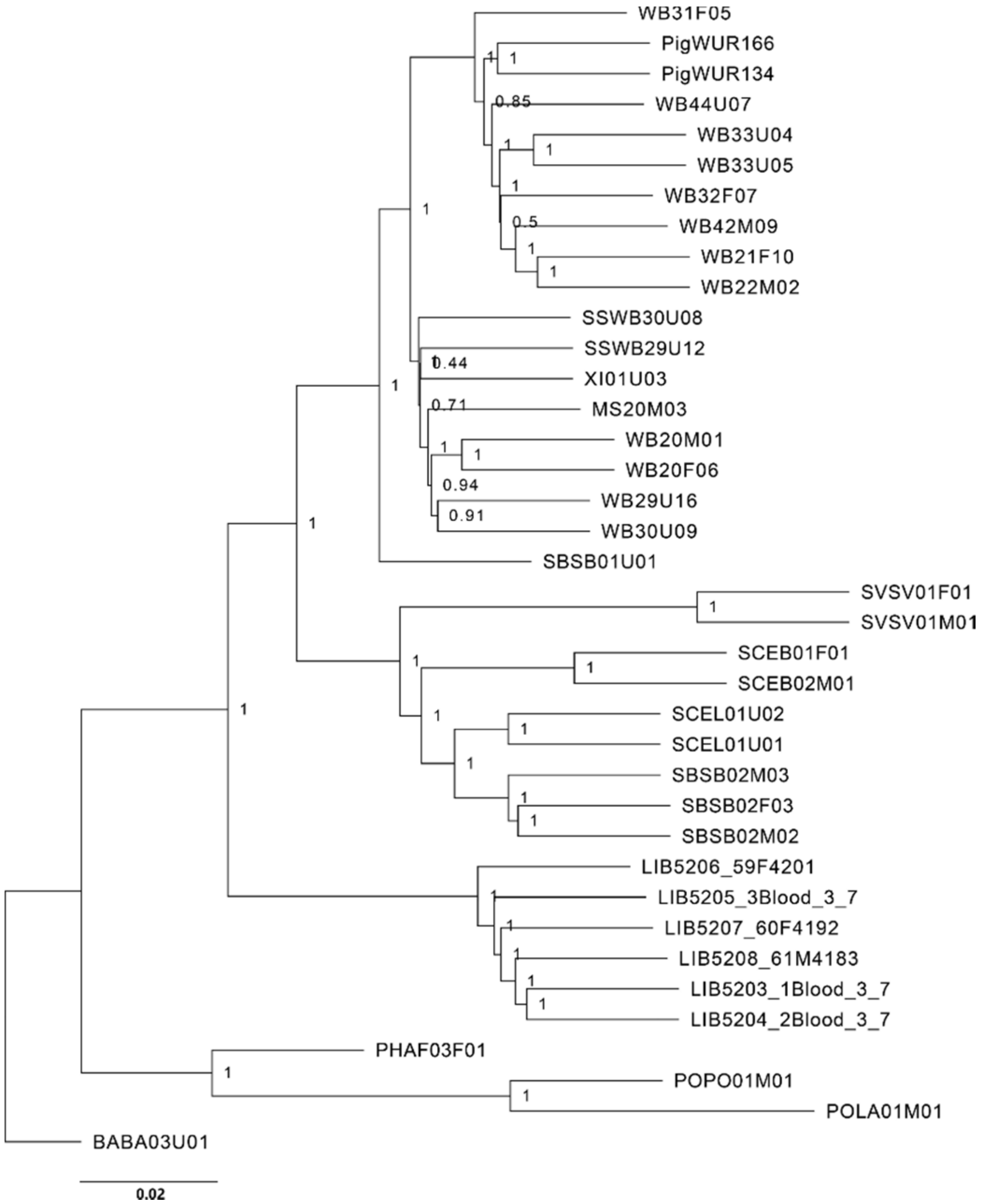

Supplementary Fig. 2

Maximum likelihood phylogenetic tree based on supertree method.

Among the 1,0142 coding sequences that are used for generating gene trees, 2,659 gene trees with highest average bootstrap values (minimum 40) were used to build a consensus species tree. The numbers on nodes represent concordance factors (ranges from 0 to 1), which represent the proportion of the genome supporting the branch. *Babyrousa babyrussa* was used as out group. See Supplementary data 1 for name abbreviation.

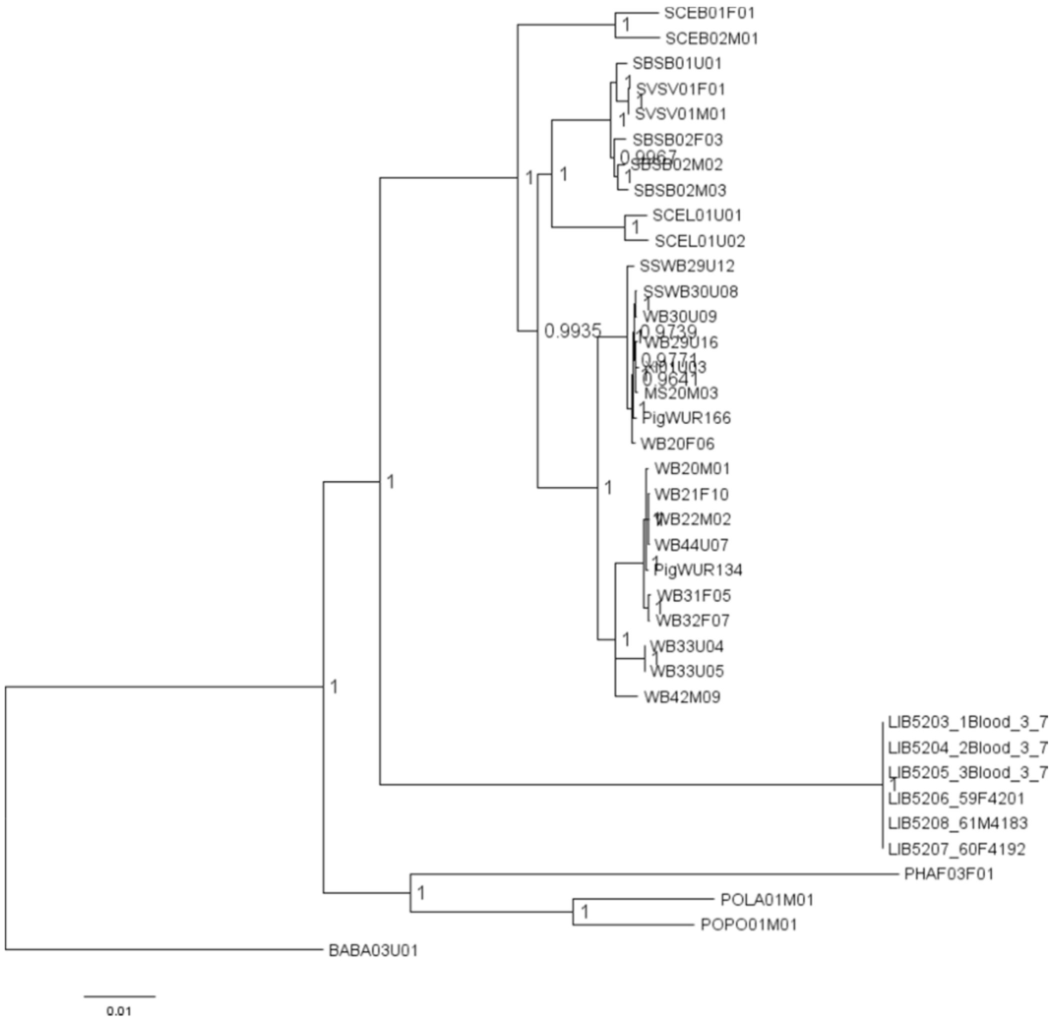

Supplementary Fig. 3

Mitochondrial phylogeny

A bayesian phylogeny of the 38 samples using complete mitochondrial genomes (16,613 bp). Pygmy hogs are the sister taxon of Sus species, with sub-Saharan suids as outgroup and the mitochondria replacement found by L.A.F Frantz et al 2014 and S.M. Funk et al 2007 is also supported[1,2]. See Supplementary data 1 for name abbreviation.

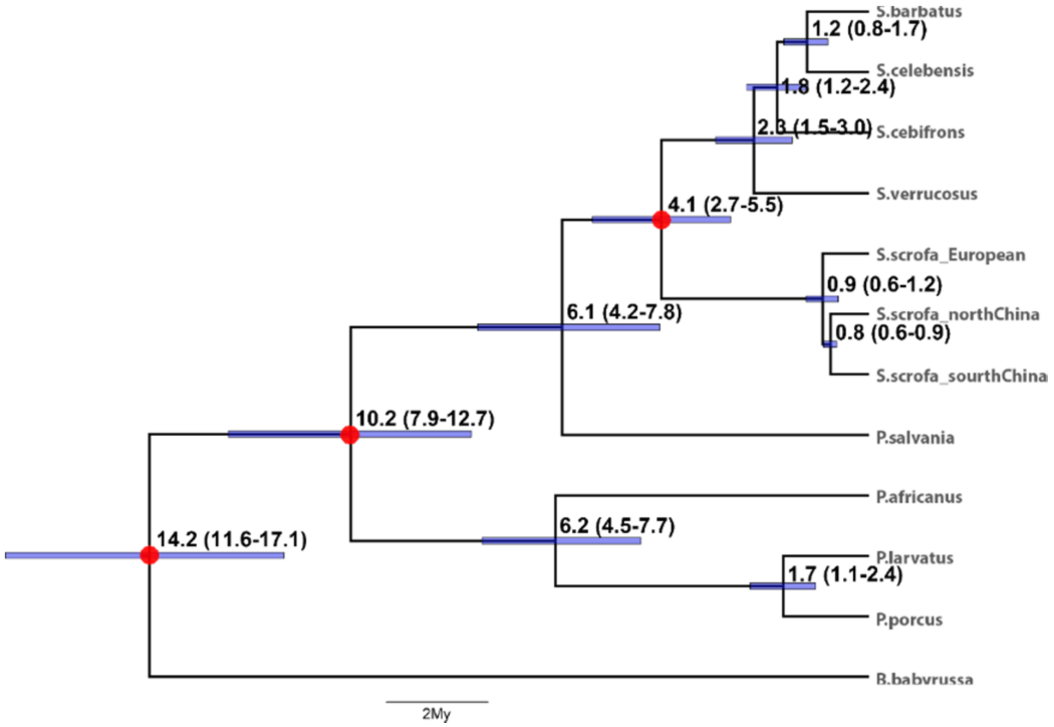

Supplementary Figure 4

Phylogenetic time tree

Species tree with mean posterior age (in millions of years). Blue bars indicated 95% confidence intervals. Red dots indicated the calibration points. We used a float prior and a maximum bound age, with a scale parameter of c=2. For the root divergence, we set the prior to (tU=2 [20 Mya], p=0.1, c=2). For MRCA of *Suinae* and *Sus*, we used the same fossil calibration as in Frantz et al. 2013 (tL=0.55 [5.5 Mya], p=0.9, c=0.5 and tL=0.2 [2 Mya], p=0.1, c=0.5, respectively).

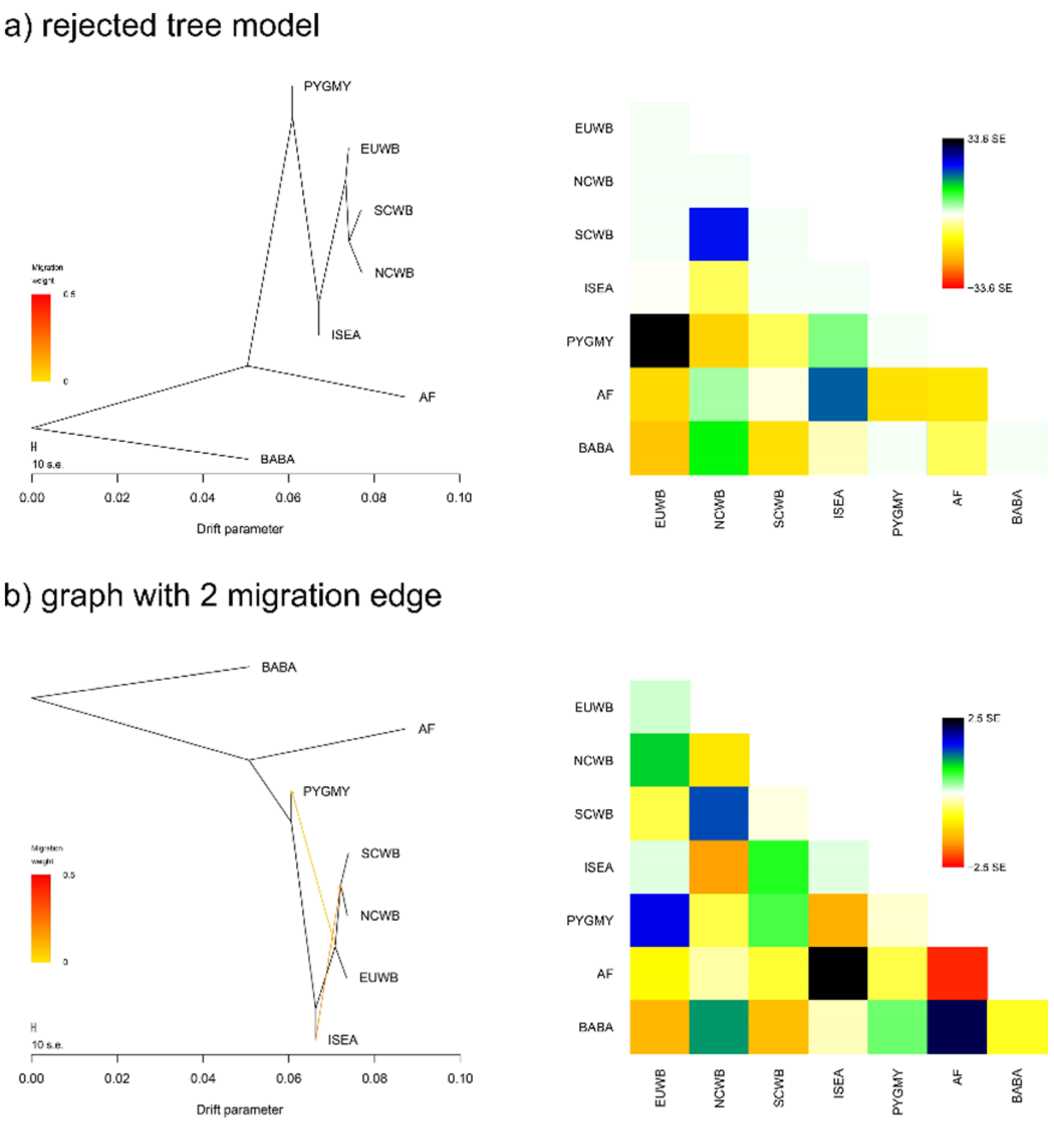

Supplementary Fig. 5

Admixture graph inferred using Treemix

a) A simple tree-like model without admixture fits the data poorly, as can be seen from the matrix of residuals between empirical and modelled allele frequency covariance on the right. b) The optimal placement of two admixture event are from the common ancestor of Eurasian wild boar to pygmy hog, as well as from ISEA Sus to common ancestor of Asian wild boar.

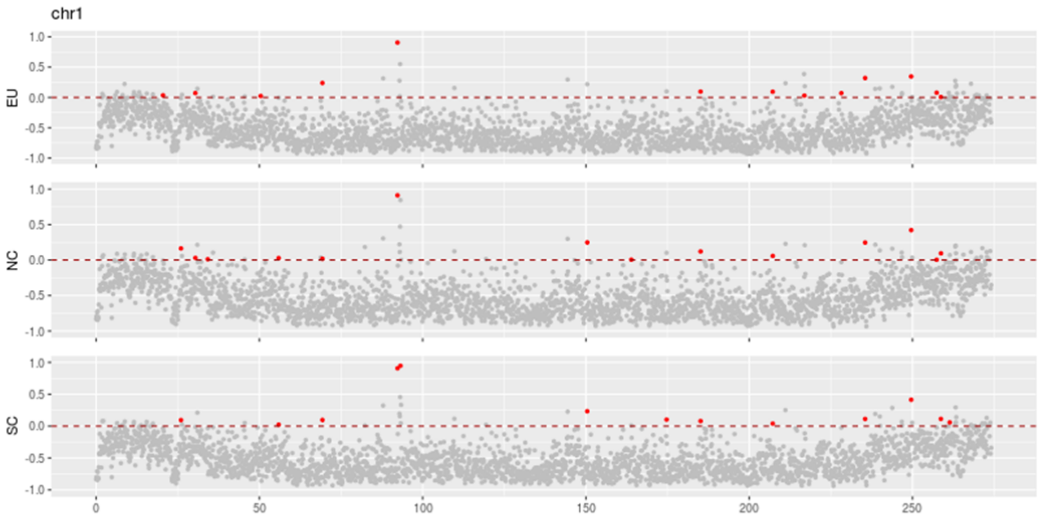

chr1
EU
NC
SC
1.0
0.5
0.0
-0.5
-1.0
0
50
100
150
200
250

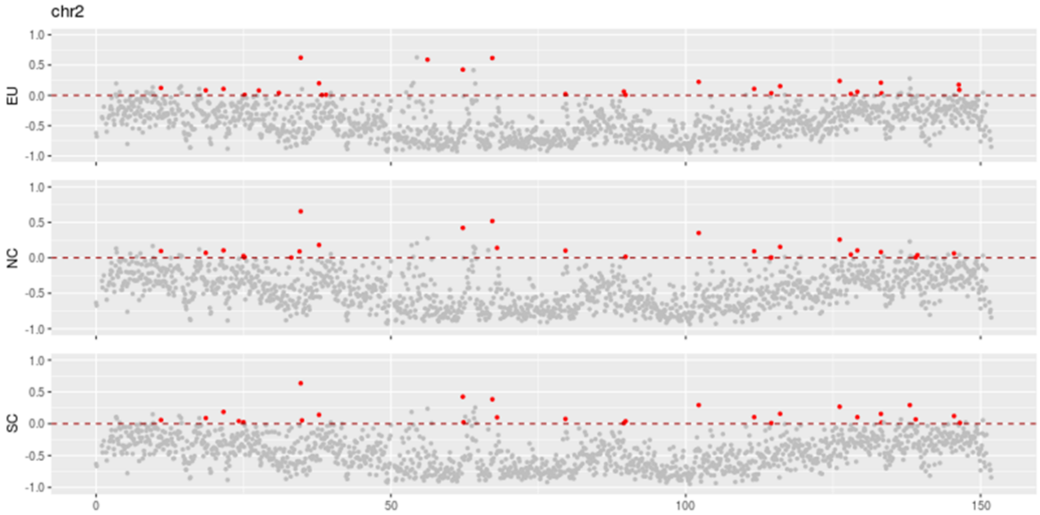

chr2
EU
NC
SC
1.0
0.5
0.0
-0.5
-1.0
0
50
100
150

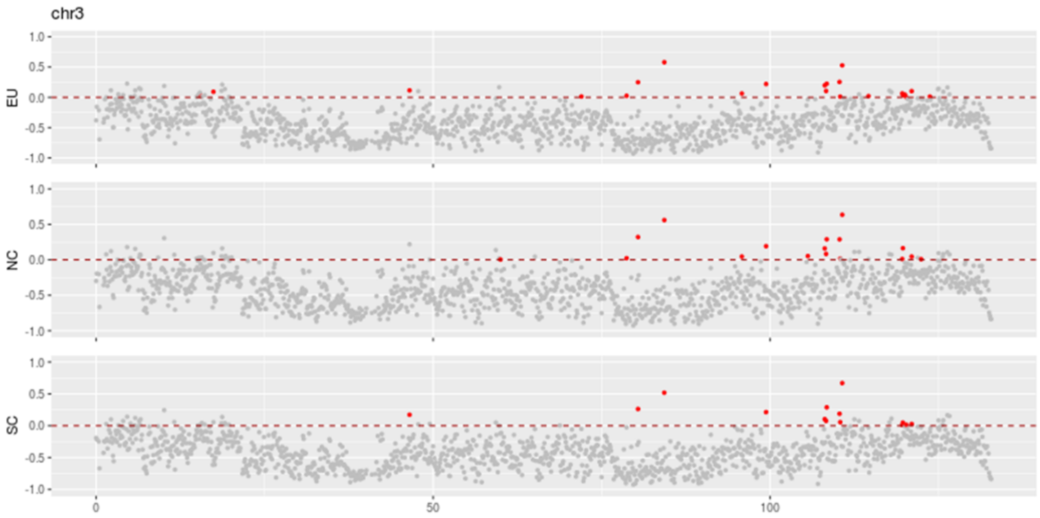
chr3
EU
NC
SC
1.0
0.5
0.0
-0.5
-1.0
0
50
100

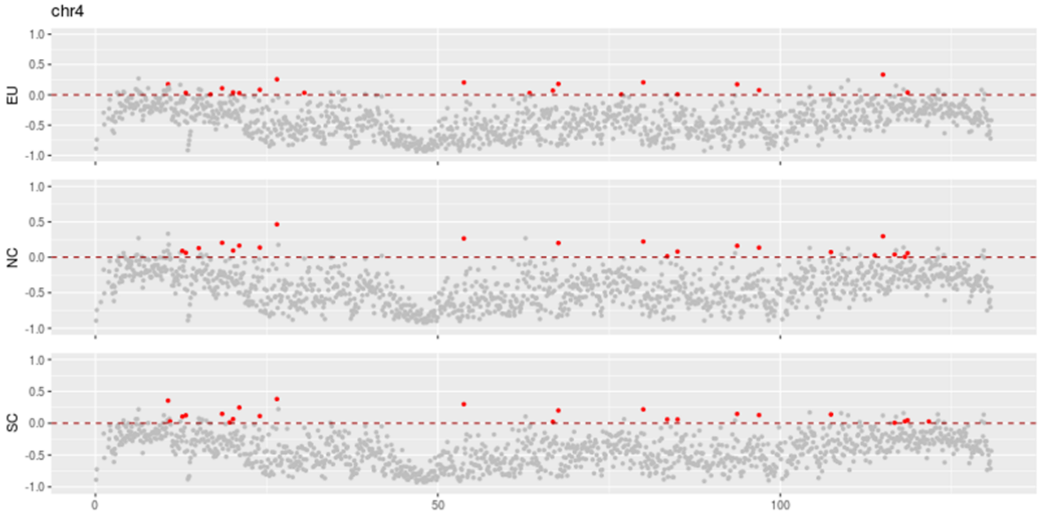
chr4
EU
NC
SC
1.0
0.5
0.0
-0.5
-1.0
0
50
100

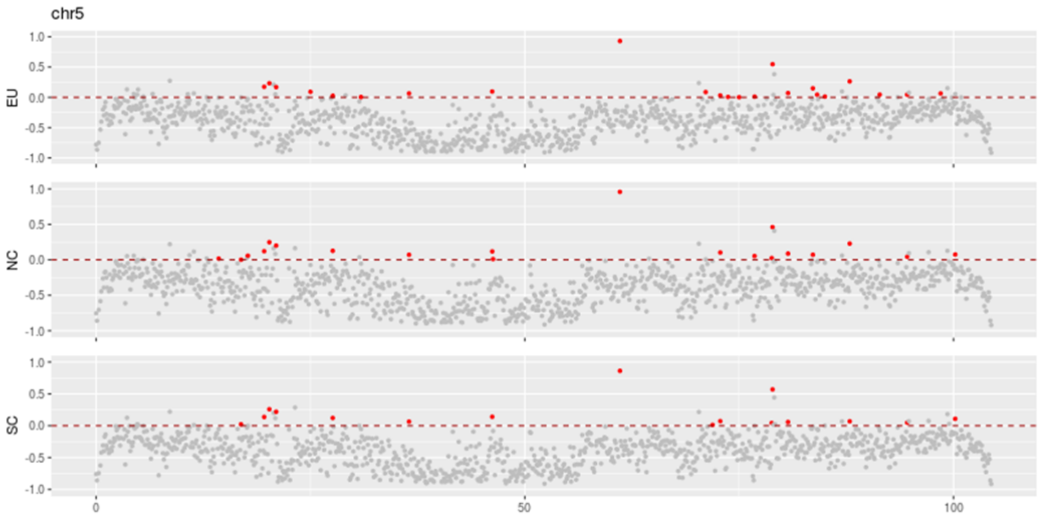
chr5
EU
NC
SC
1.0
0.5
0.0
-0.5
-1.0
0
50
100

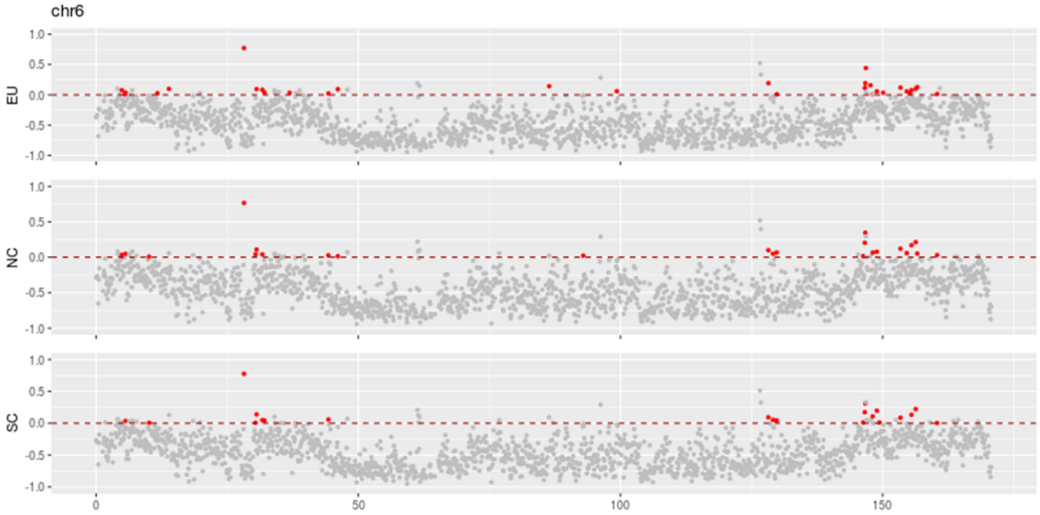
chr6
EU
NC
SC
1.0
0.5
0.0
-0.5
-1.0
0
50
100
150

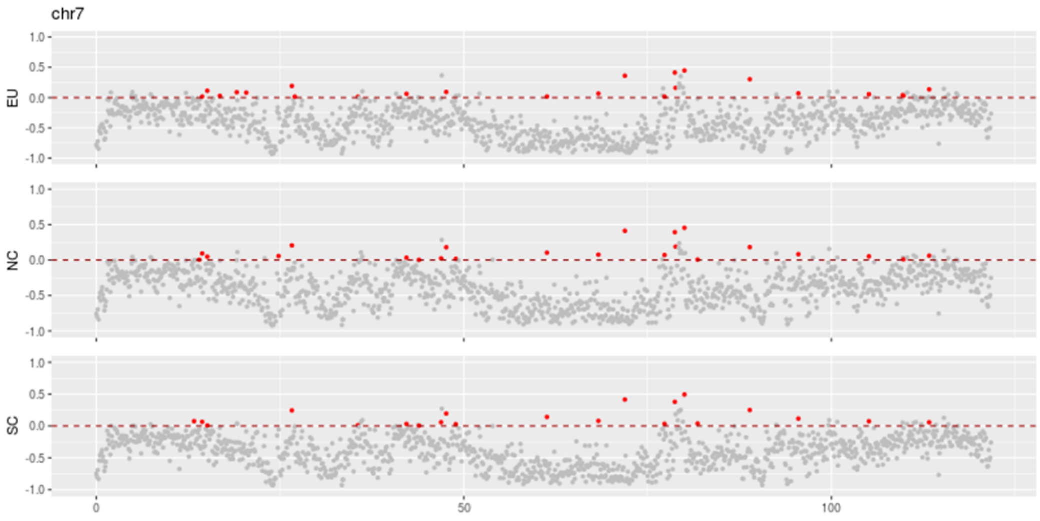
chr7
EU
NC
SC
1.0
0.5
0.0
-0.5
-1.0
0
50
100

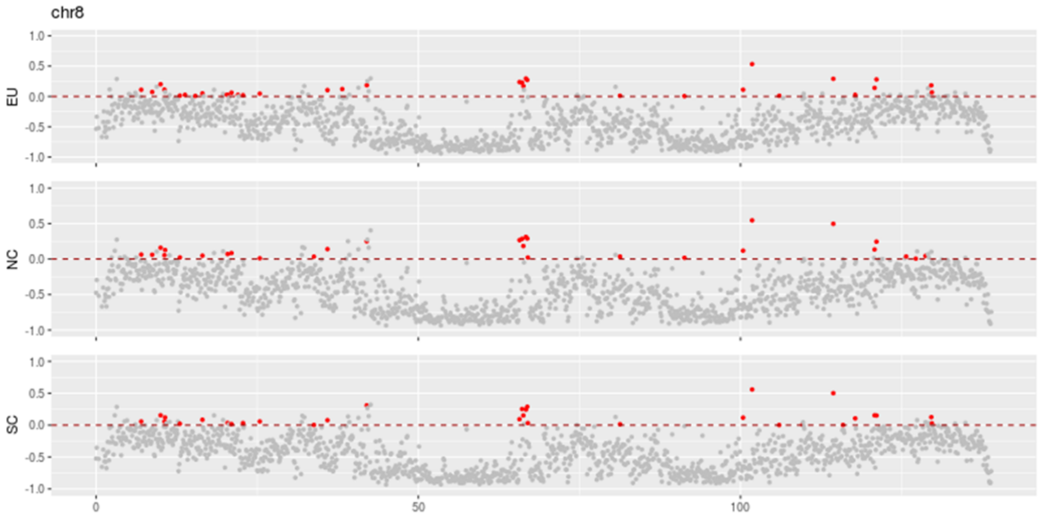
chr8
EU
NC
SC
1.0
0.5
0.0
-0.5
-1.0
0
50
100

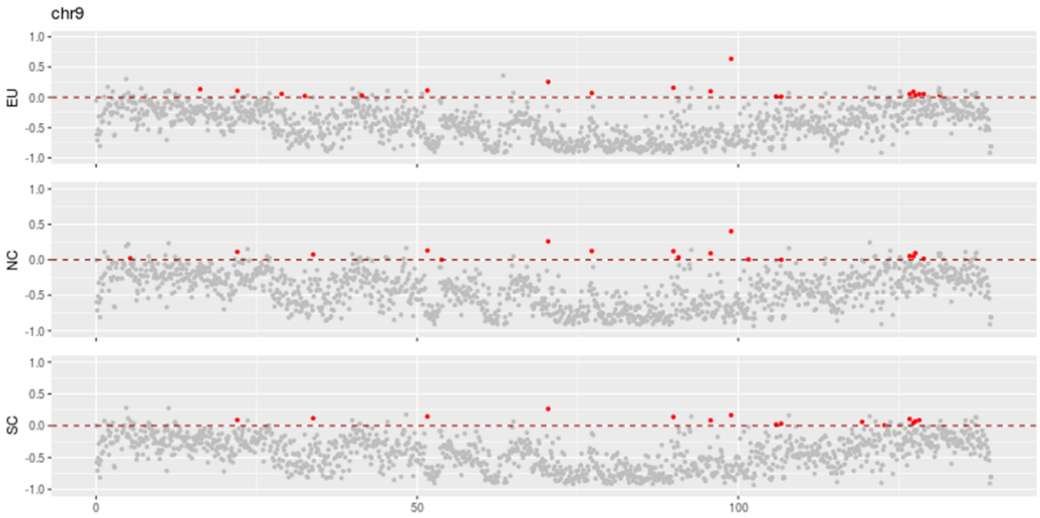
chr9
EU
NC
SC
1.0
0.5
0.0
-0.5
-1.0
0
50
100

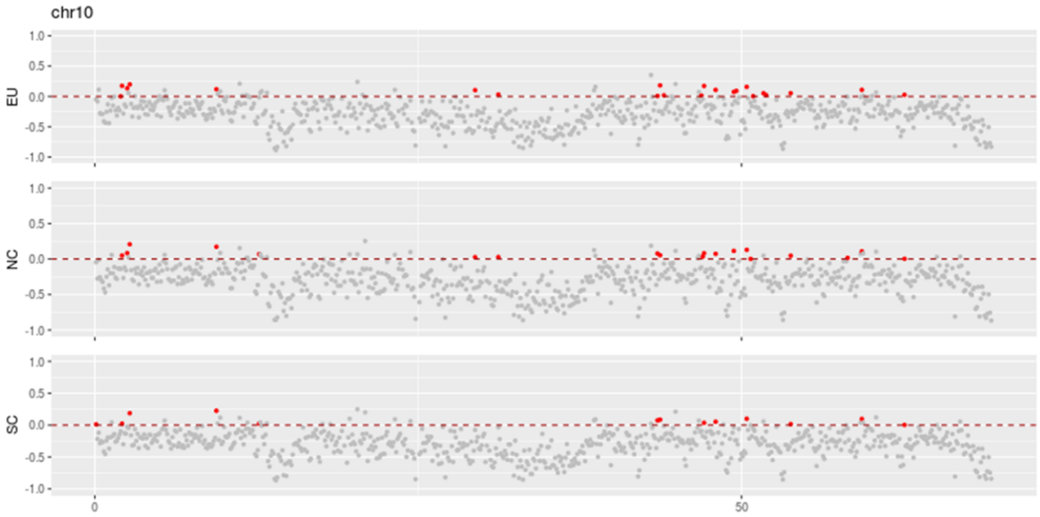
chr10
EU
NC
SC
1.0
0.5
0.0
-0.5
-1.0
0
50

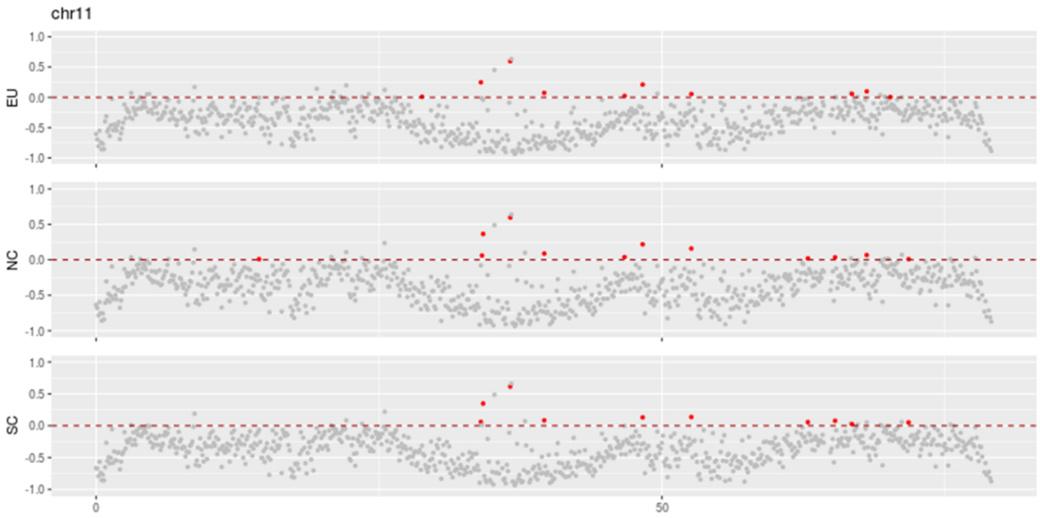
chr11
EU
NC
SC
1.0
0.5
0.0
-0.5
-1.0
0
50

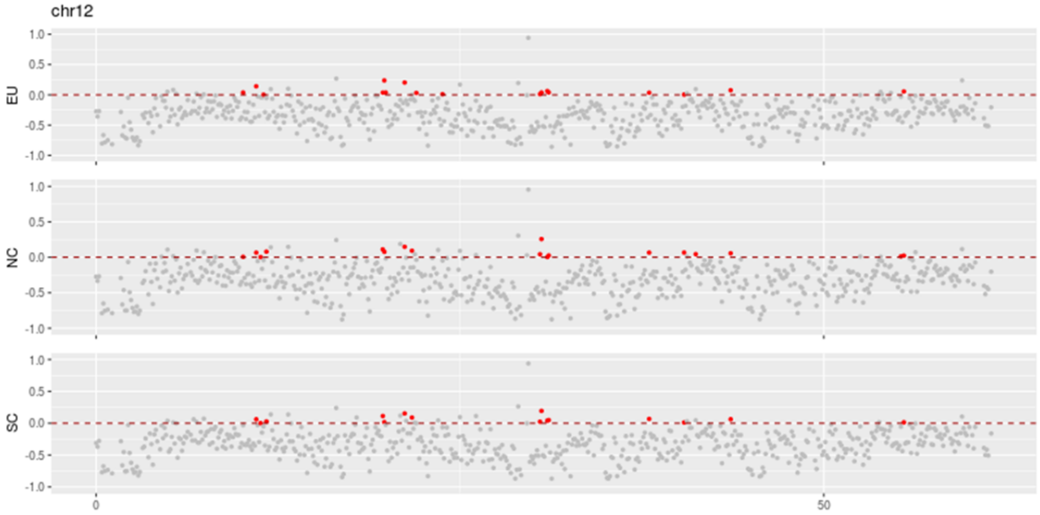
chr12
EU
NC
SC
1.0
0.5
0.0
-0.5
-1.0
0
50

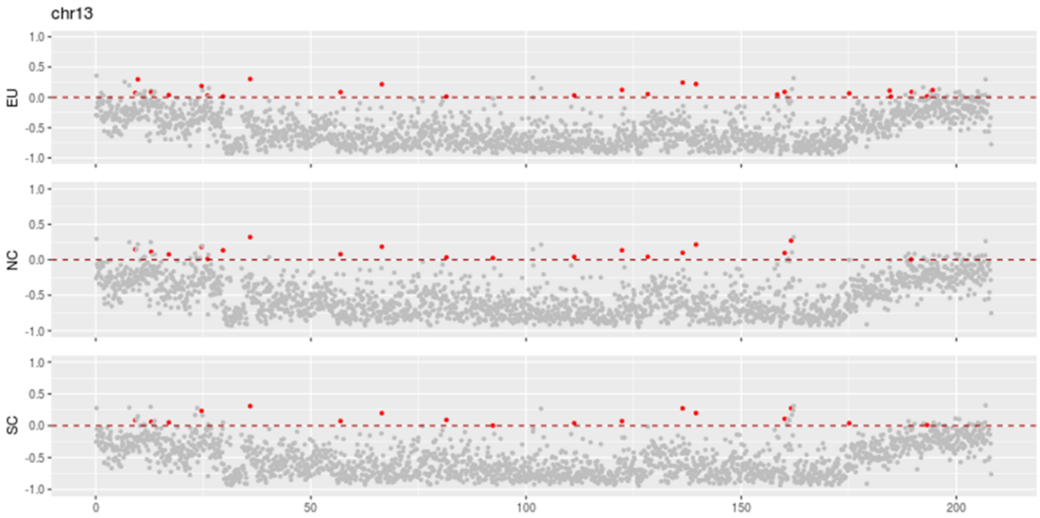
chr13
EU
NC
SC
1.0
0.5
0.0
-0.5
-1.0
0
50
100
150
200

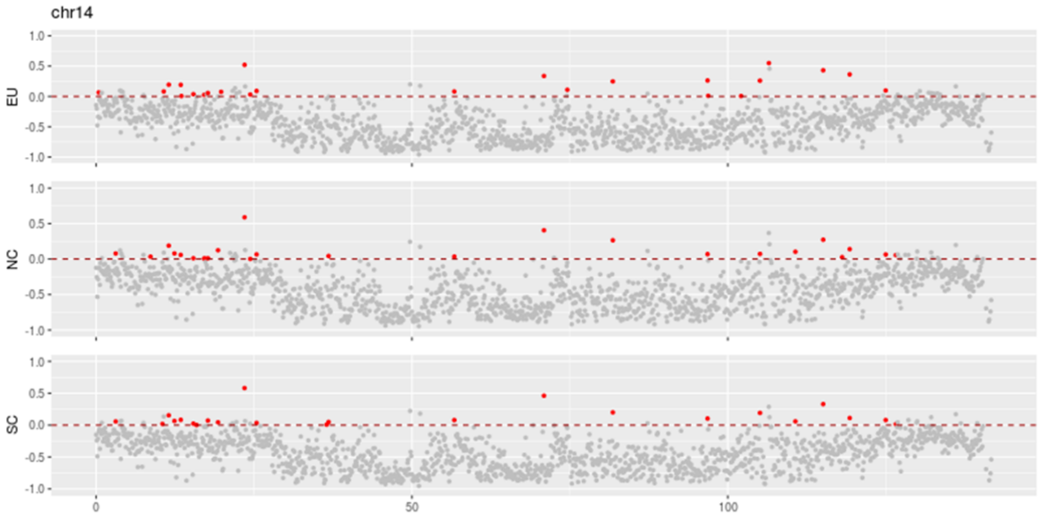
chr14
EU
NC
SC
1.0
0.5
0.0
-0.5
-1.0
0
50
100

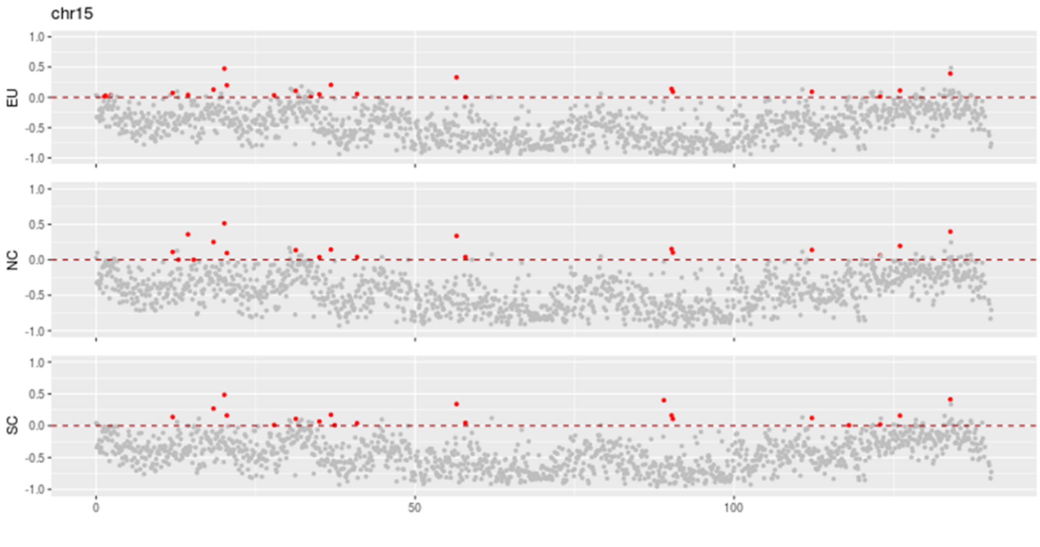
chr15
EU
NC
SC
1.0
0.5
0.0
-0.5
-1.0
0
50
100

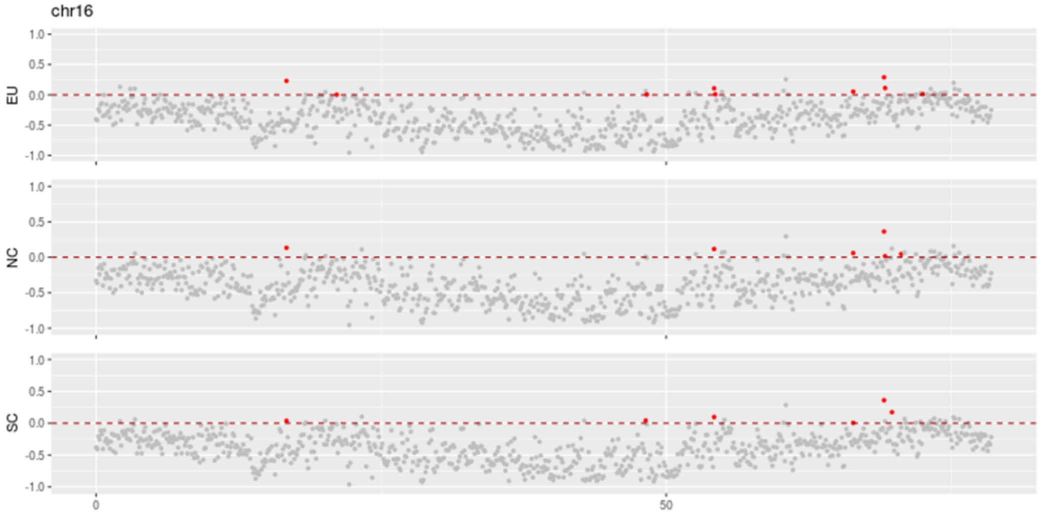
chr16
EU
NC
SC
1.0
0.5
0.0
-0.5
-1.0
0
50

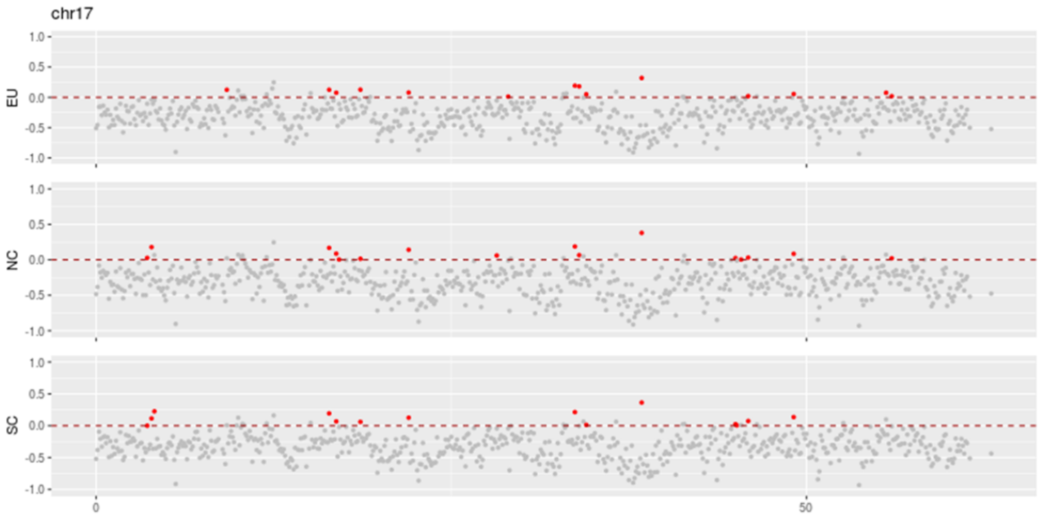
chr17
EU
NC
SC
1.0
0.5
0.0
-0.5
-1.0
0
50

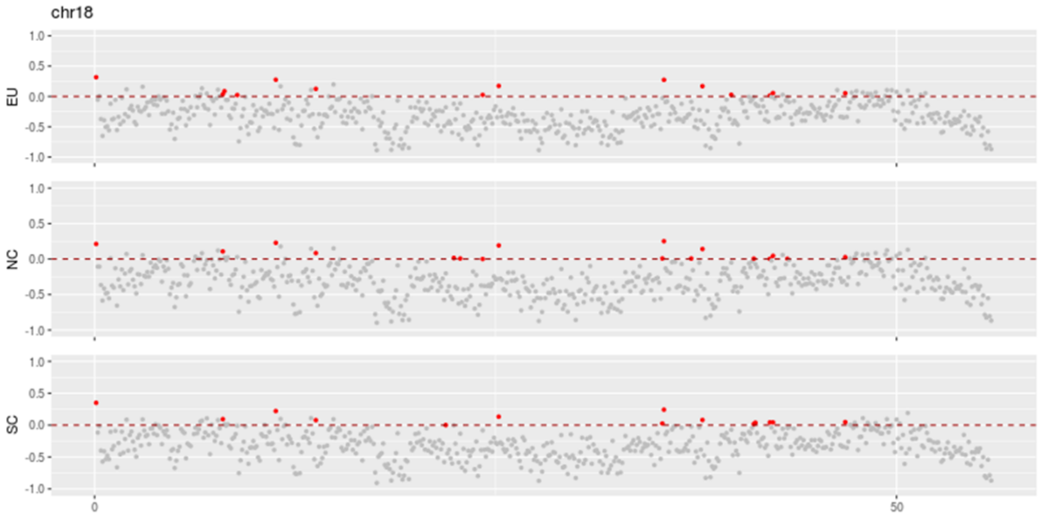
chr18
EU
NC
SC
1.0
0.5
0.0
-0.5
-1.0
0
50

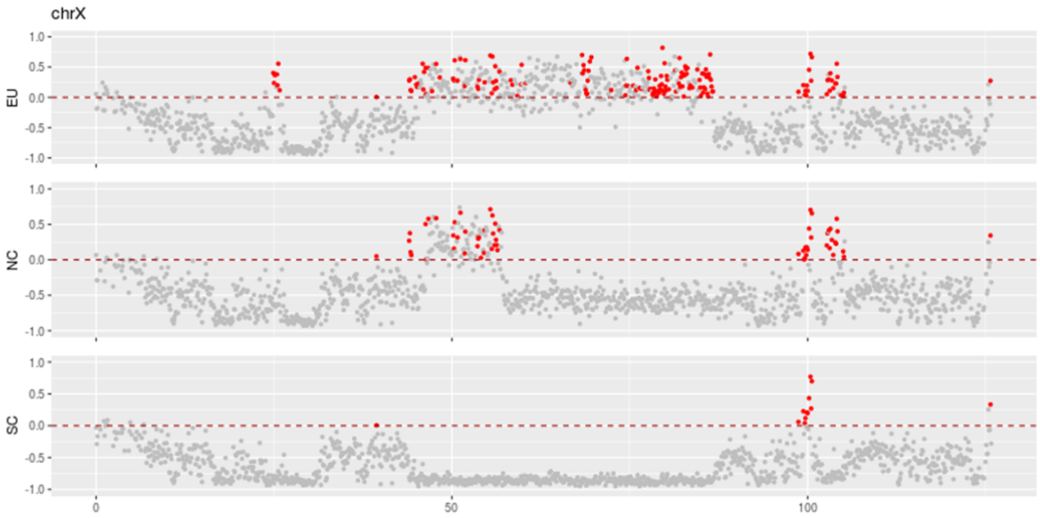

Supplementary Fig. 6

The D-statistic and fd for testing introgression for 100-kb windows on each chromosome with the tree topology (((ISEA, Pygmy hog), X), warthog), X=European wildboar (EU), Northern China wildboar (NC) or Southern China wildboar (SC). We also calculated DNA sequence divergence (dxy) between X and pygmy hog for each window and color the windows red whose dxy is in the lower 25% distribution of all the windows and D-value>0. Chr = chromosome, ISEA = Island of South east Asia pigs

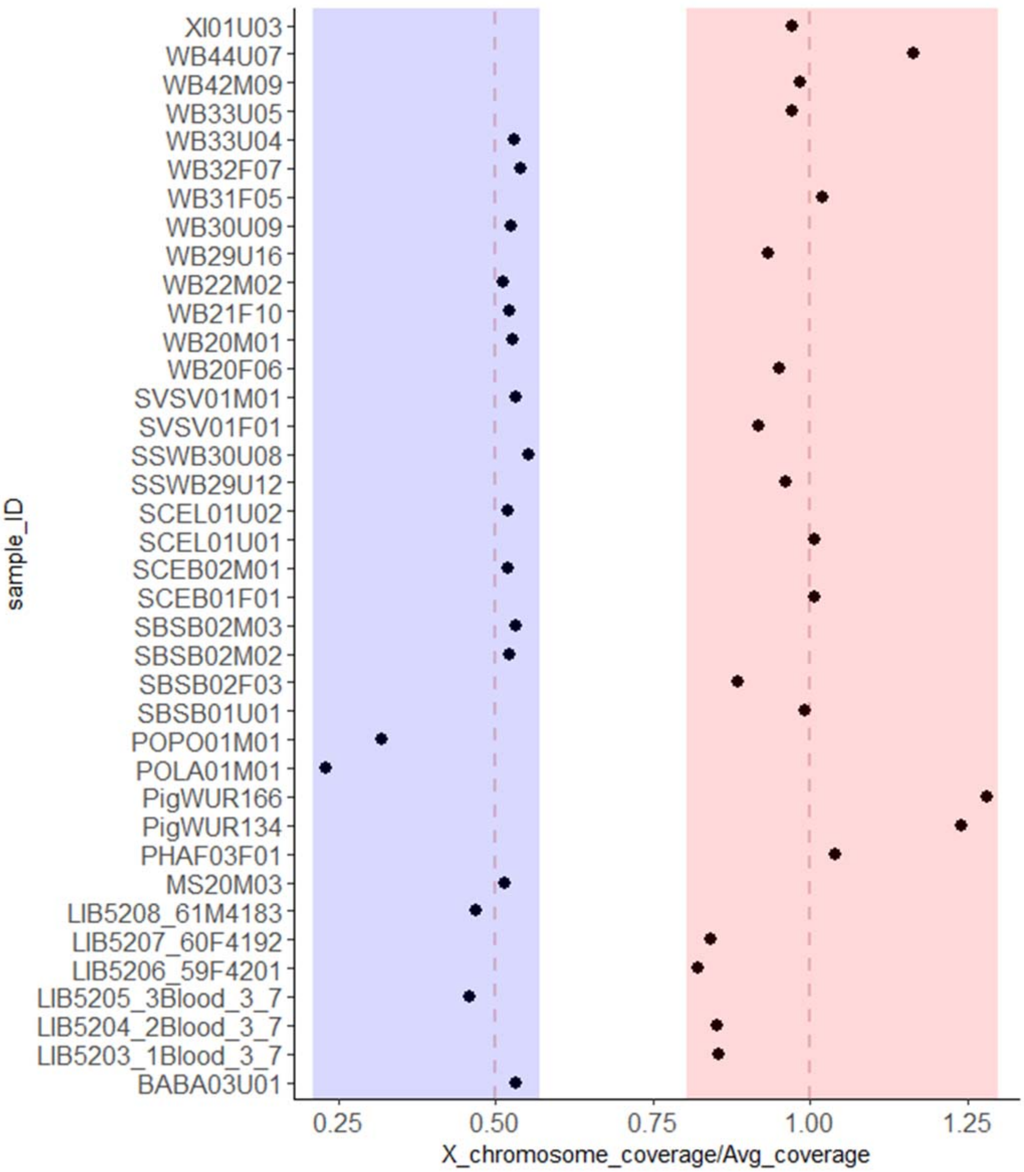

Supplementary Fig. 7

Observed fraction of X chromosome read depth compared to the average read depth of autosomes in all individuals in this study.

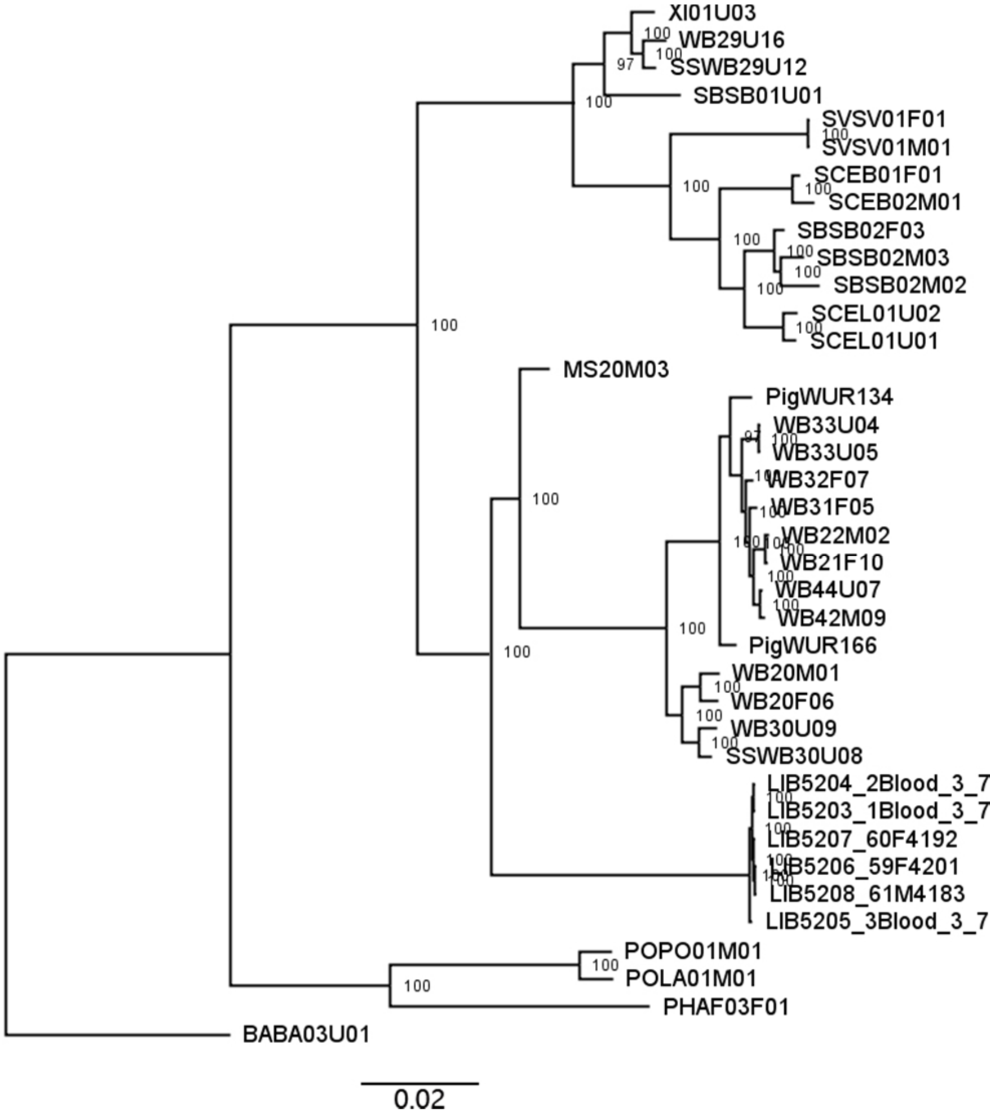

Supplementary Fig. 8

X chromosome topology

The maximum likelihood tree of the whole X chromosome (Optimization Likelihood: -223197633.0). The topology of the whole X chromosome tree was inconsistent with the topology of autosomal phylogenetic trees (Supplementary Fig. 1&2). The numbers represent node support inferred from 100 non-parametric bootstrap repetitions. B.babyrussa is the outgroup. See Supplementary data 1 for name abbreviation.

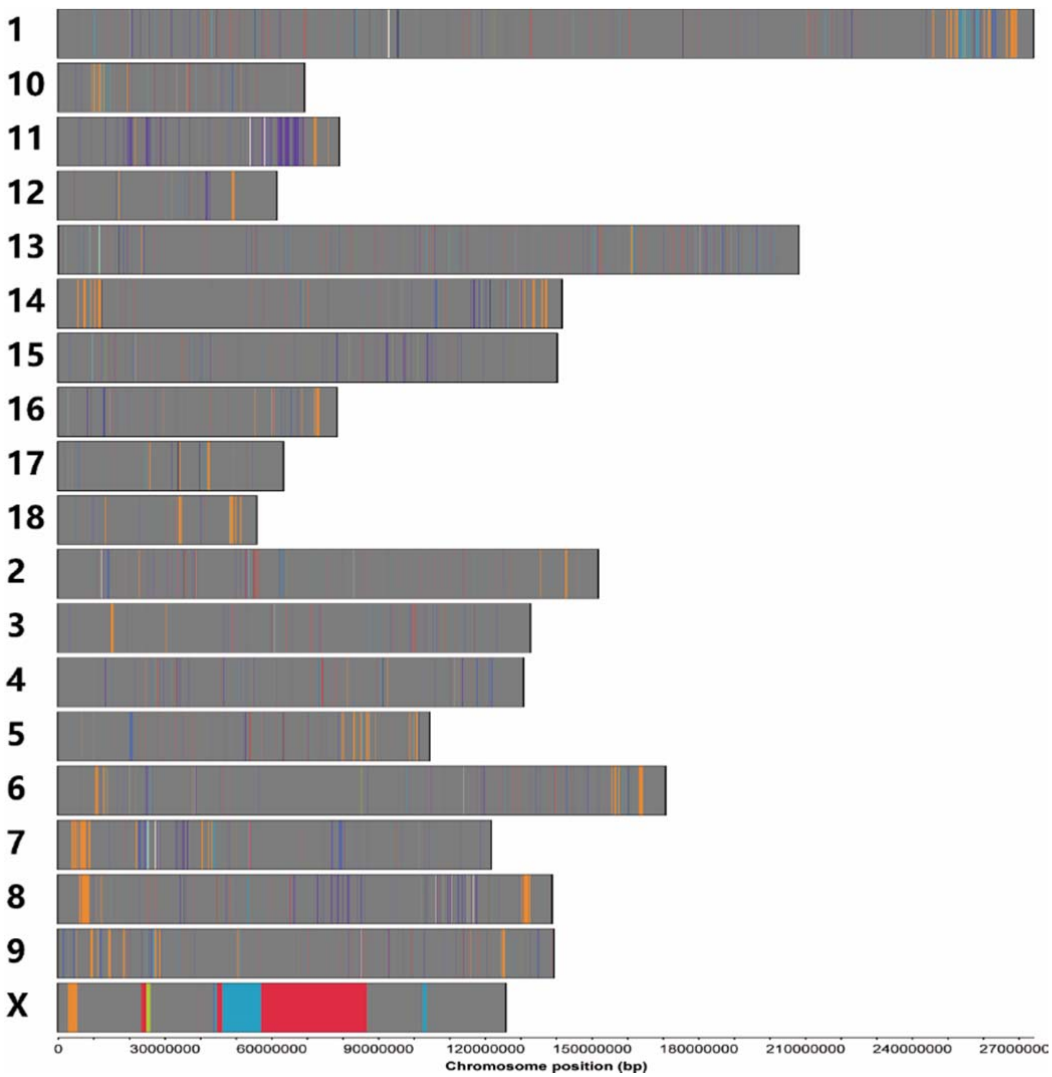

Supplementary Fig. 9

Distribution of chromosomal segments supporting the 12 most frequent tree topologies assigned by SAGUARO to segments along the autosomes and the X chromosome. The numbers given next to phylogenies indicate chromosome IDs. See Supplementary data 3 for further details.

123

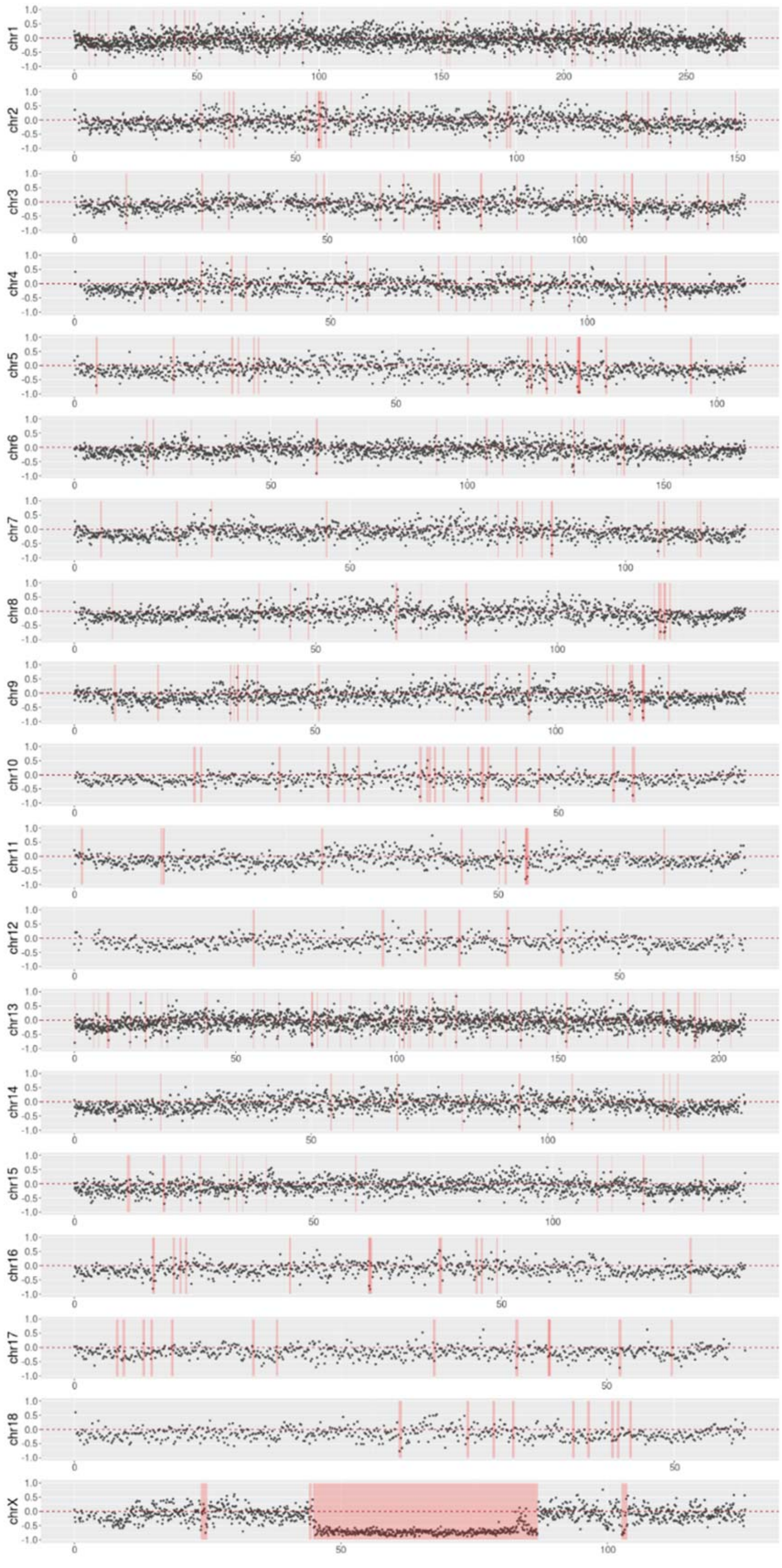
chr1
chr2
chr3
chr4
chr5
chr6
chr7
chr8
chr9
chr10
chr11
chr12
chr13
chr14
chr15
chr16
chr17
chr18
chrX

Supplementary Fig. 10

The D-statistic for testing introgression for 100-kb windows on chromosome 1-18 and X. For the tree topology (((ISEA, EUWB), pygmy hog), warthog). The ABBA-BABA method and its related statistics compute the excess of shared derived mutation between two taxa compared to a control not connected by gene flow to the others. The outgroup allows mutations to be polarized. A mean D value of 0 is expected if the two taxa are not connected by gene flow. Red shaded areas show the introgression regions inferred by Saguaro using the same quadruplet. ISEA=Island of South Asia pigs, EUWB = European wild boar.

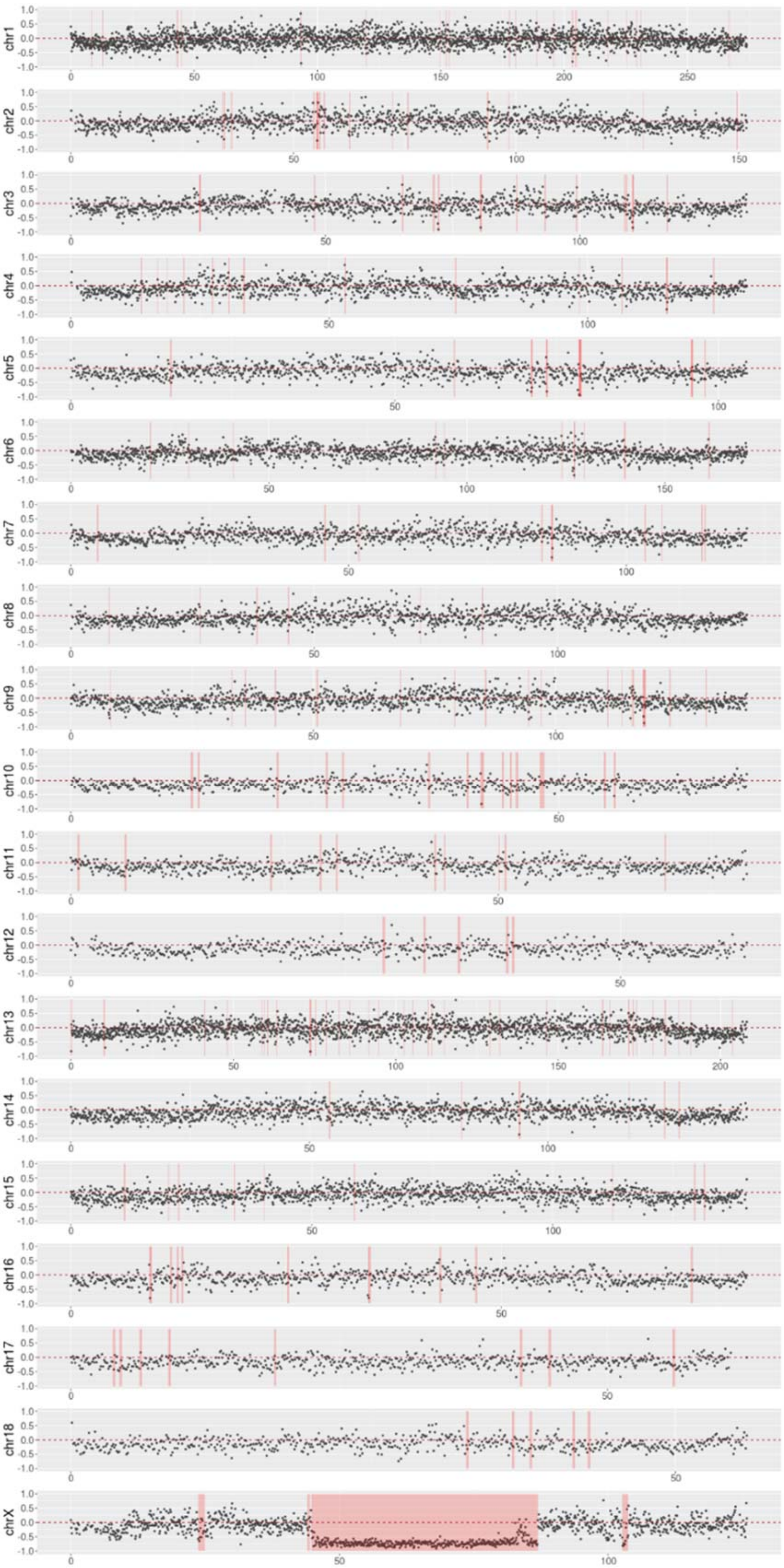
chr1
chr2
chr3
chr4
chr5
chr6
chr7
chr8
chr9
chr10
chr11
chr12
chr13
chr14
chr15
chr16
chr17
chr18
chrX

Supplementary Fig. 11

The D-statistic for testing introgression for 100-kb windows on chromosome 1-18 and X. For the tree topology (((ISEA, EUD), pygmy hog), warthog). Red shaded areas show the introgression regions inferred by Saguaro using the same quadruplet. ISEA=Island of South Asia pigs, EUD = European domesticated pig.

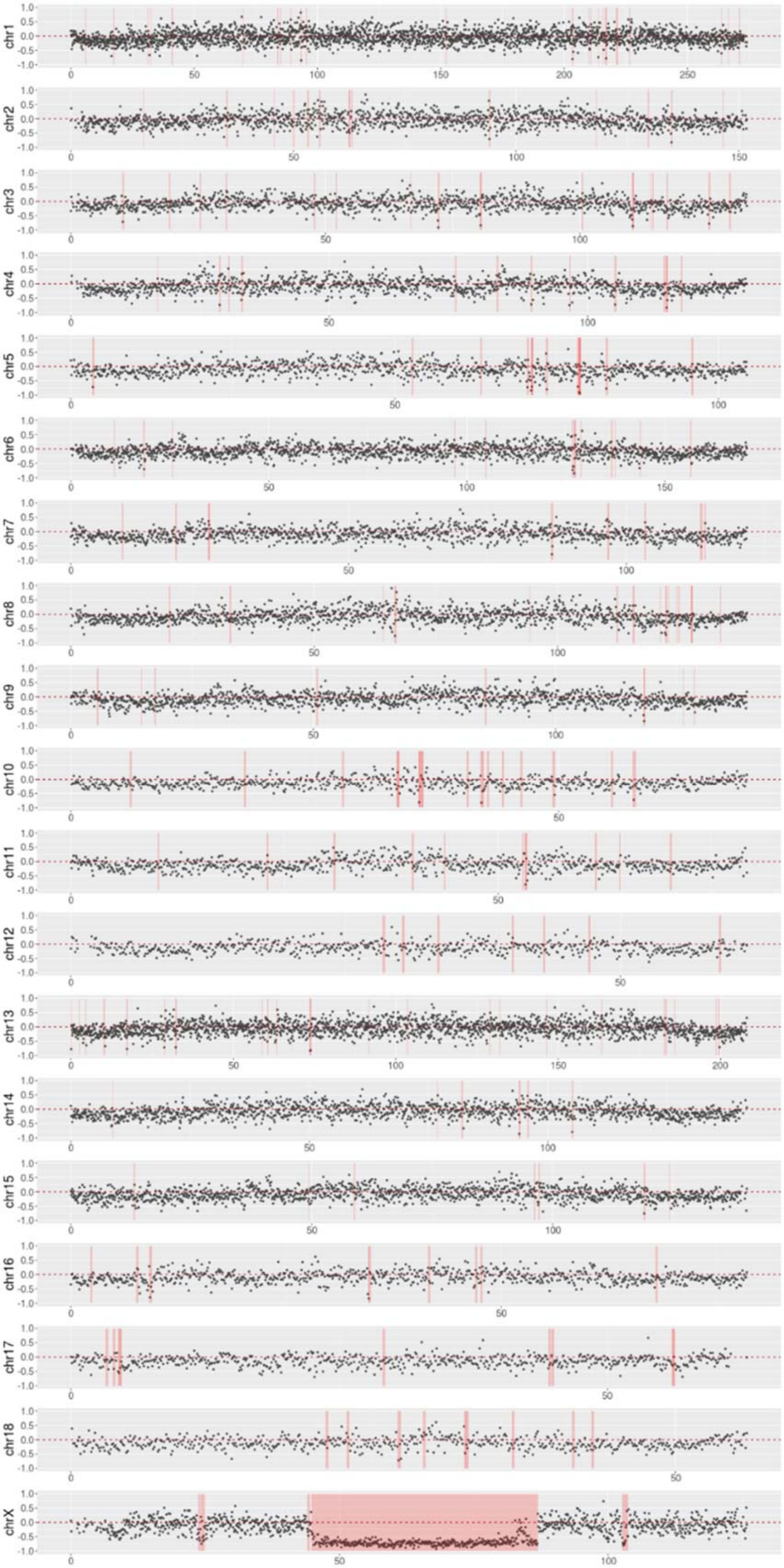
chr1
chr2
chr3
chr4
chr5
chr6
chr7
chr8
chr9
chr10
chr11
chr12
chr13
chr14
chr15
chr16
chr17
chr18
chrX

Supplementary Fig. 12

The D-statistic for testing introgression for 100-kb windows on chromosome 1-18 and X. For the tree topology (((ISEA, NCWB), pygmy hog), warthog). Red shaded areas show the introgression regions inferred by Saguaro using the same quadruplet. ISEA=Island of South Asia pigs, NCWB = North Chinese wild boar.

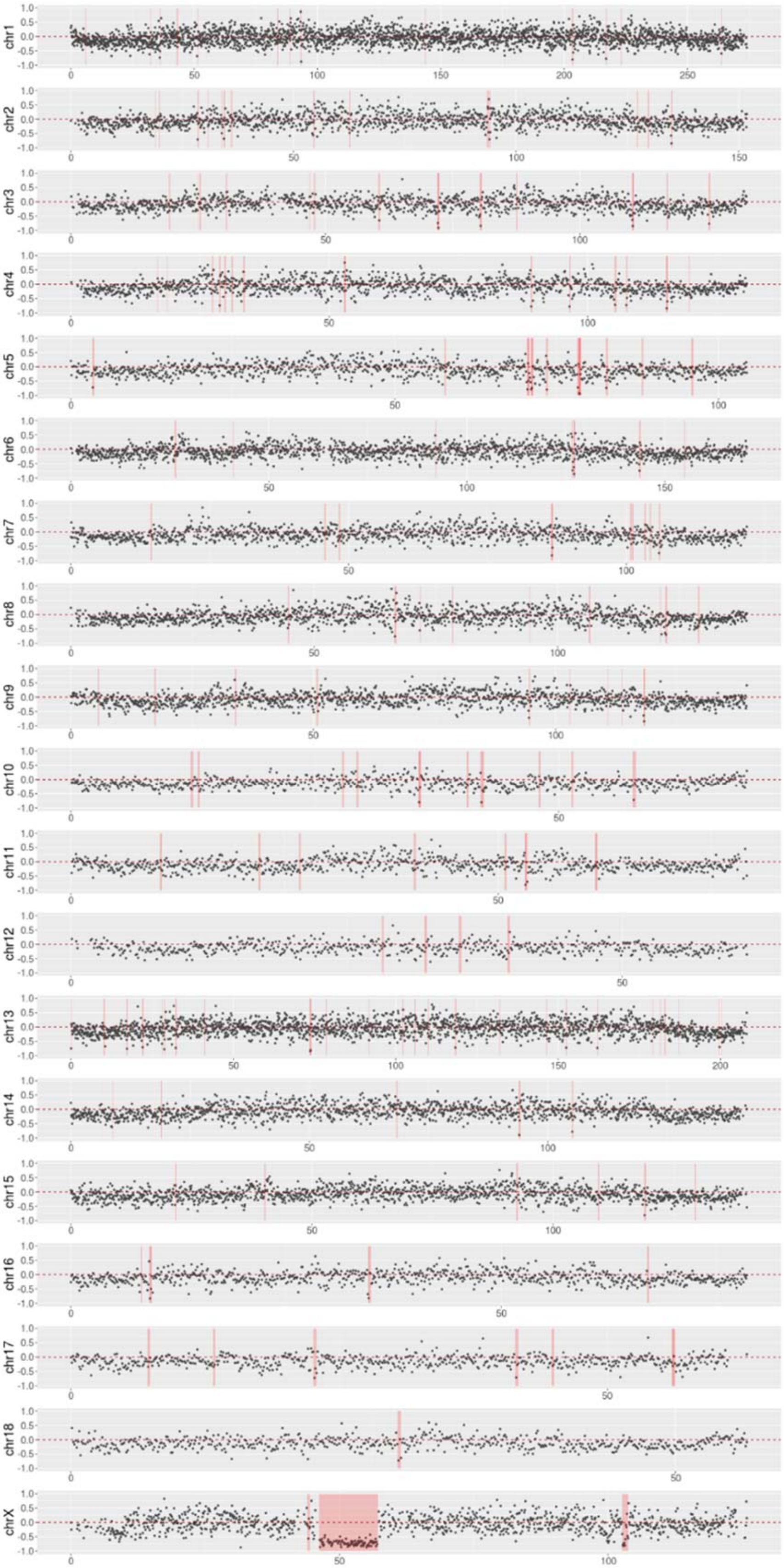
chr1
chr2
chr3
chr4
chr5
chr6
chr7
chr8
chr9
chr10
chr11
chr12
chr13
chr14
chr15
chr16
chr17
chr18
chrX

Supplementary Fig. 13

The D-statistic for testing introgression for 100-kb windows on chromosome 1-18 and X. For the tree topology (((ISEA, NCD), pygmy hog), warthog). Red shaded areas show the introgression regions inferred by Saguaro using the same quadruplet. ISEA=Island of South Asia pigs, NCD = North Chinese domesticated pig.

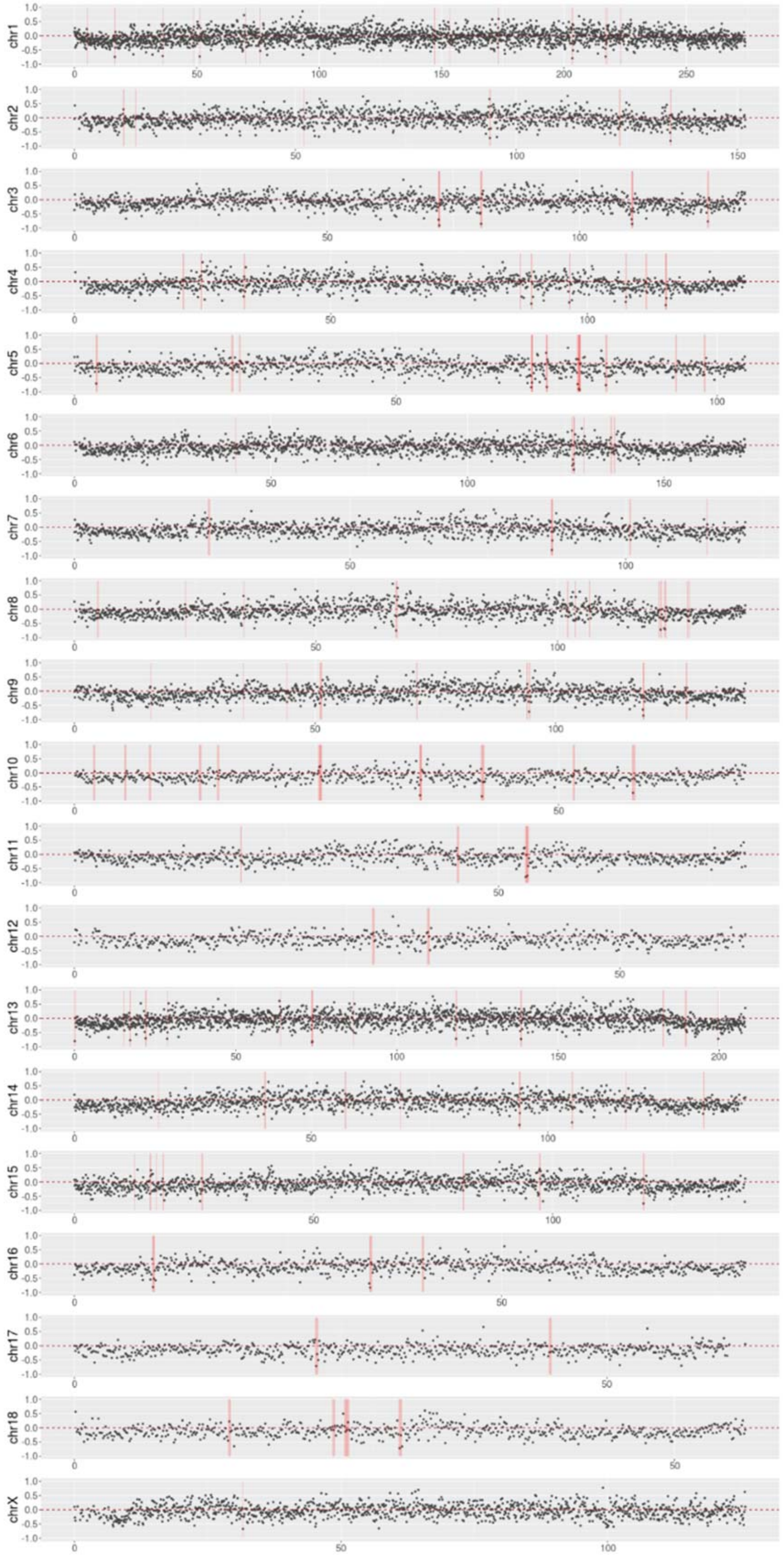
chr1
chr2
chr3
chr4
chr5
chr6
chr7
chr8
chr9
chr10
chr11
chr12
chr13
chr14
chr15
chr16
chr17
chr18
chrX

Supplementary Fig. 14

The D-statistic for testing introgression for 100-kb windows on chromosome 1-18 and X. For the tree topology (((ISEA, SCWB), pygmy hog), warthog). Red shaded areas show the introgression regions inferred by Saguaro using the same quadruplet. ISEA=Island of South Asia pigs, SCWB = South Chinese wild boar.

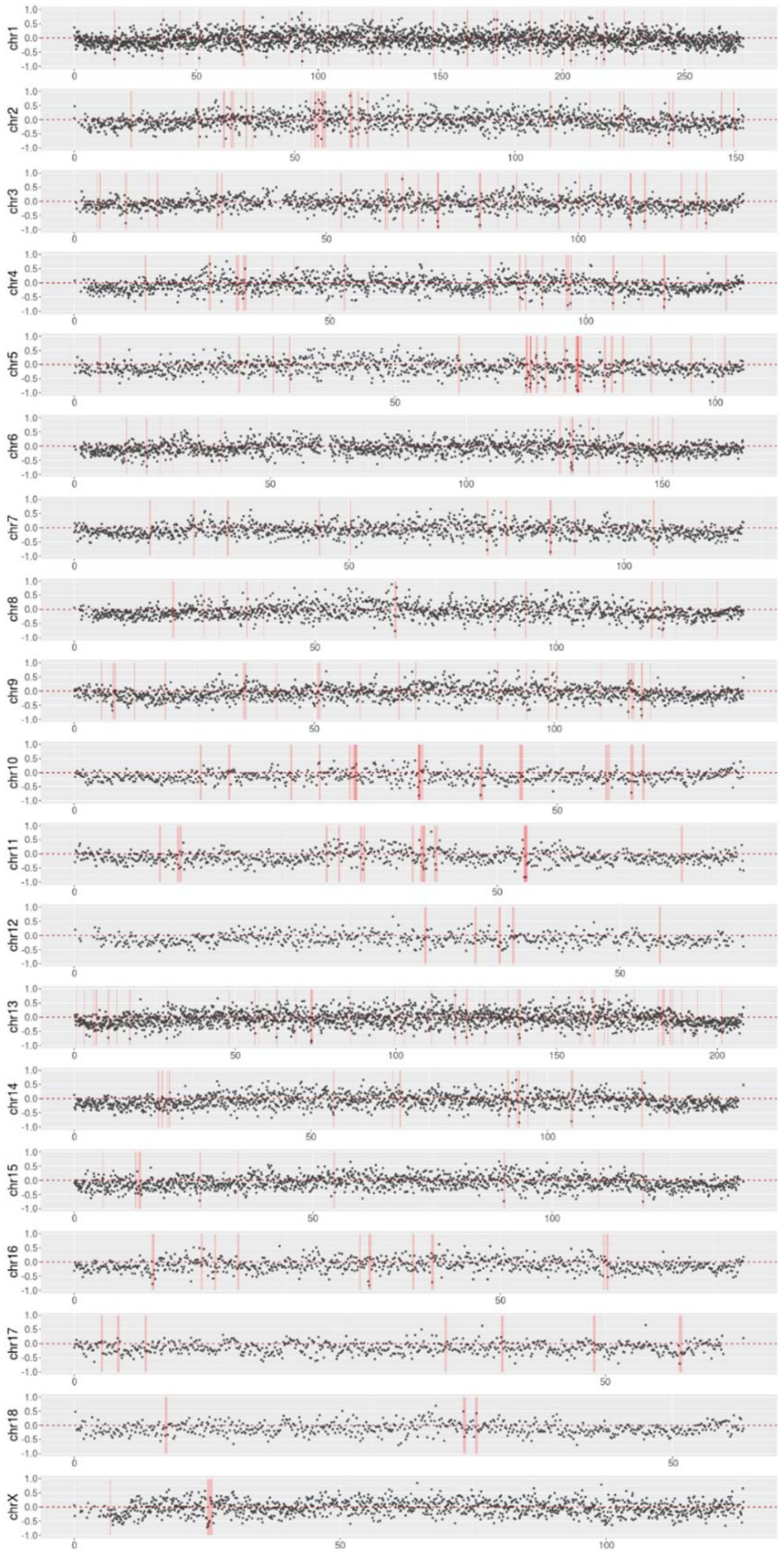
chr1
chr2
chr3
chr4
chr5
chr6
chr7
chr8
chr9
chr10
chr11
chr12
chr13
chr14
chr15
chr16
chr17
chr18
chrX

Supplementary Fig. 15

The D-statistic for testing introgression for 100-kb windows on chromosome 1-18 and X. For the tree topology (((ISEA, SCD), pygmy hog), warthog). Red shaded areas show the introgression regions inferred by Saguaro using the same quadruplet. ISEA=Island of South Asia pigs, SCD = South Chinese domesticated pig.

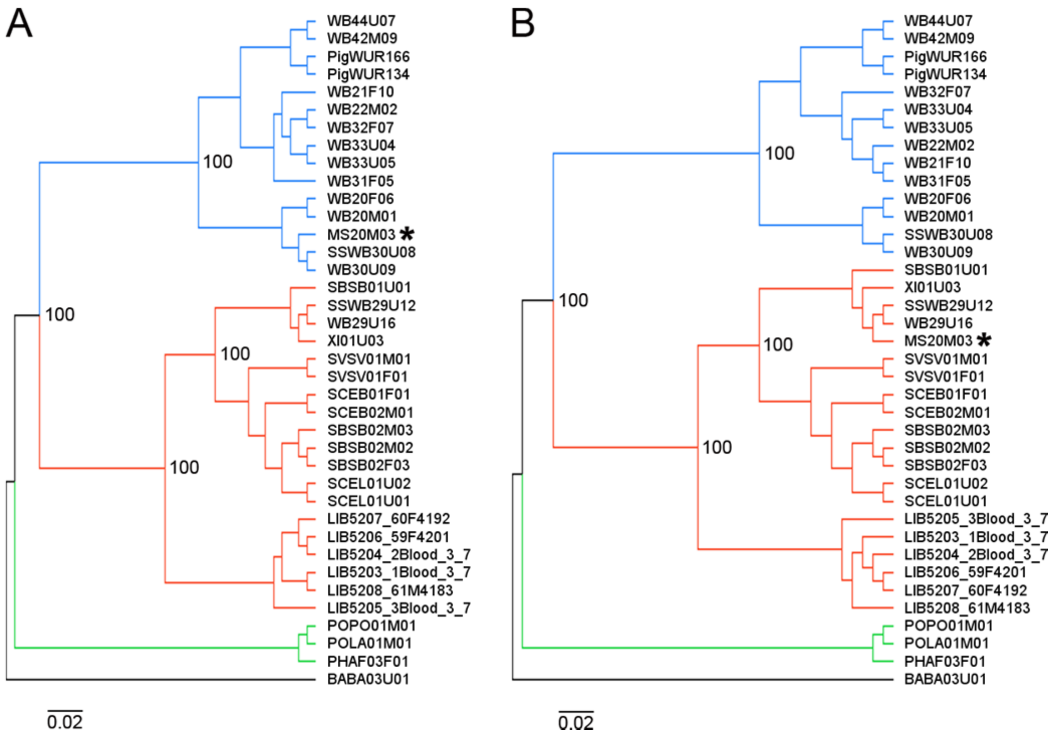

Supplementary Fig. 16

X chromosome topology for the two introgression regions.

Maximum likelihood tree for the two genealogical discordant regions on chromosome X. A. Maximum likelihood tree using the first discordant region spanning 52.0~57.8Mb on X chromosome (Optimization Likelihood: -18385084.9). B. Maximum likelihood tree built using the second discordant region spanning 57.8~91.5Mb on X chromosome (Optimization Likelihood: -50166590.1). Meishan (North China domestic breed) was marked with asterisk (*) to highlight that this breed has different haplotypes in the two regions. See Supplementary data 1 for name abbreviation. The numbers at the nodes represent bootstrap values based on 100 non-parametric bootstrap replications

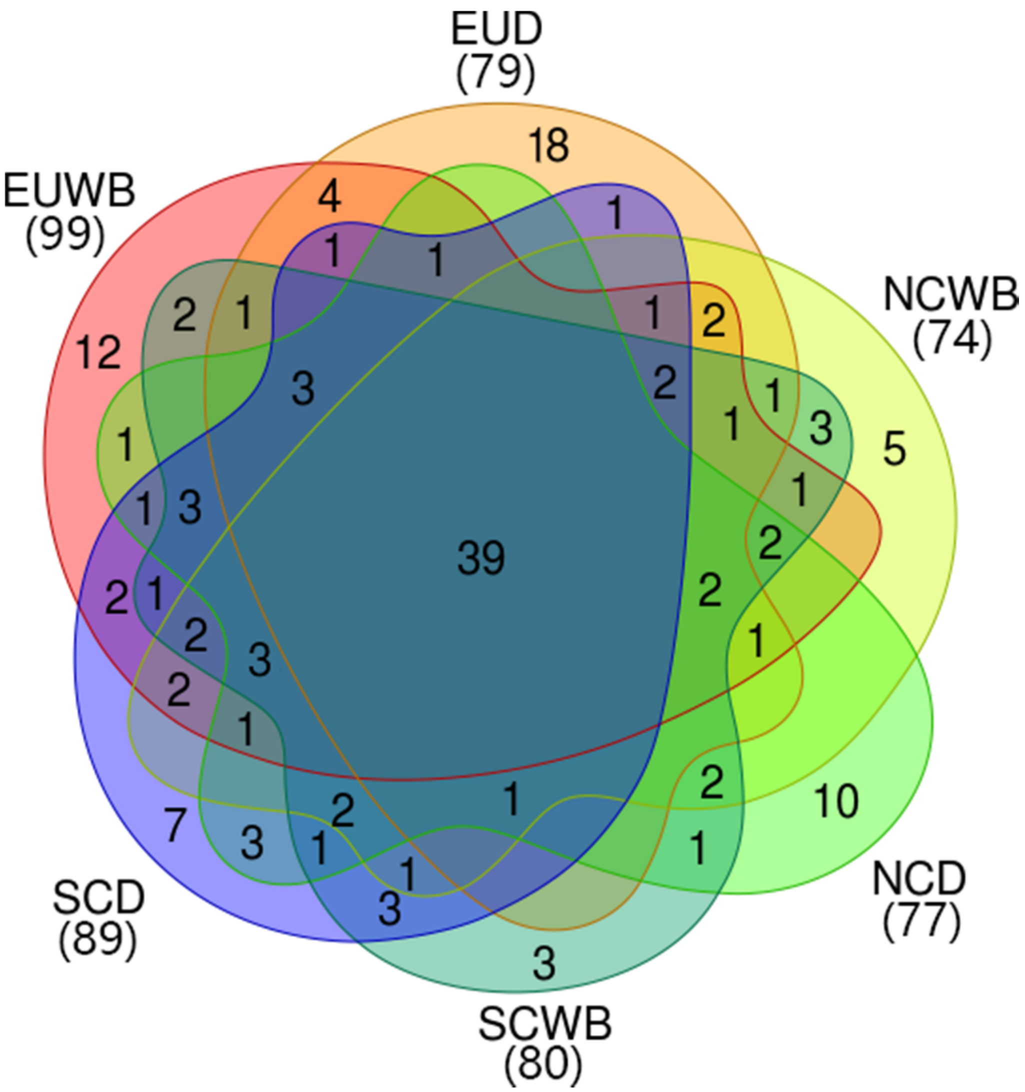

Supplementary Fig. 17

The Venn diagram shows shared autosomal segments with ‘ghost’ introgression signal in sliding-window ABBA-BABA analysis among different swine populations. For European pigs, we calculated the average D-value for the clear ‘ghost’ introgression region on X chromosome and any autosomal windows with D-value lower than this will be regarded as introgression region. Numbers in parentheses refer to the total amount of introgression windows in each population. SCWB = South Chinese wild boar, SCD = South Chinese domesticated pig, NCWB = North Chinese wild boar, NCD = North Chinese domesticated pig, EUWB = European wild boar, EUD = European domesticated pig.

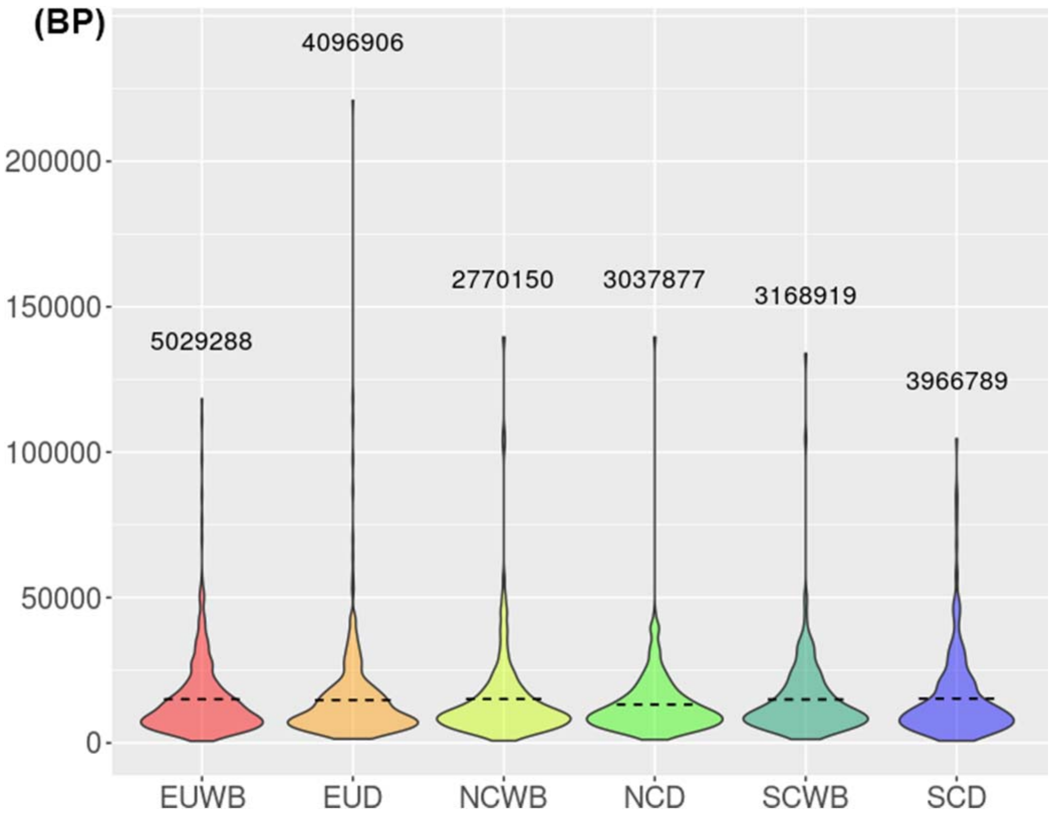

Supplementary Fig. 18

Violin plot representing the length of autosomal ‘ghost’ introgression region in different population inferred by Saguaro. Numbers on top of each violin are the total length of introgression region. Dash lines indicated the average length. SCWB = South Chinese wild boar, SCD = South Chinese domesticated pig, NCWB = North Chinese wild boar, NCD = North Chinese domesticated pig, EUWB = European wild boar, EUD = European domesticated pig.

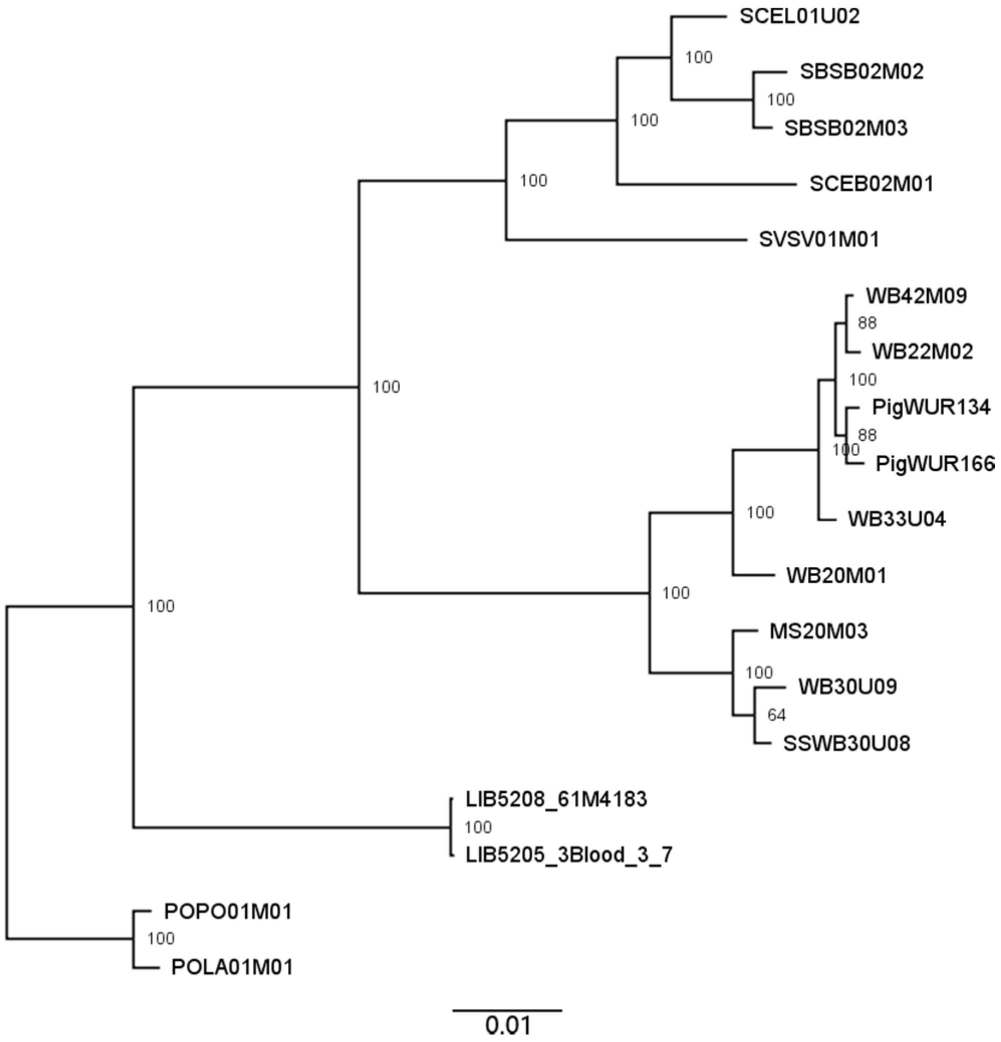

Supplementary Fig. 19

Y chromosome topology

The maximum likelihood tree of the non- recombining Y chromosome (Optimization Likelihood: -21216029.3). The topology of Y chromosome tree was consistent with the topology of autosomal phylogenetic trees (Supplementary Fig. 1&2). The numbers represent node support inferred from 100 non-parametric bootstrap repetitions. B.babyrussa is the outgroup. See Supplementary data 1 for name abbreviation.

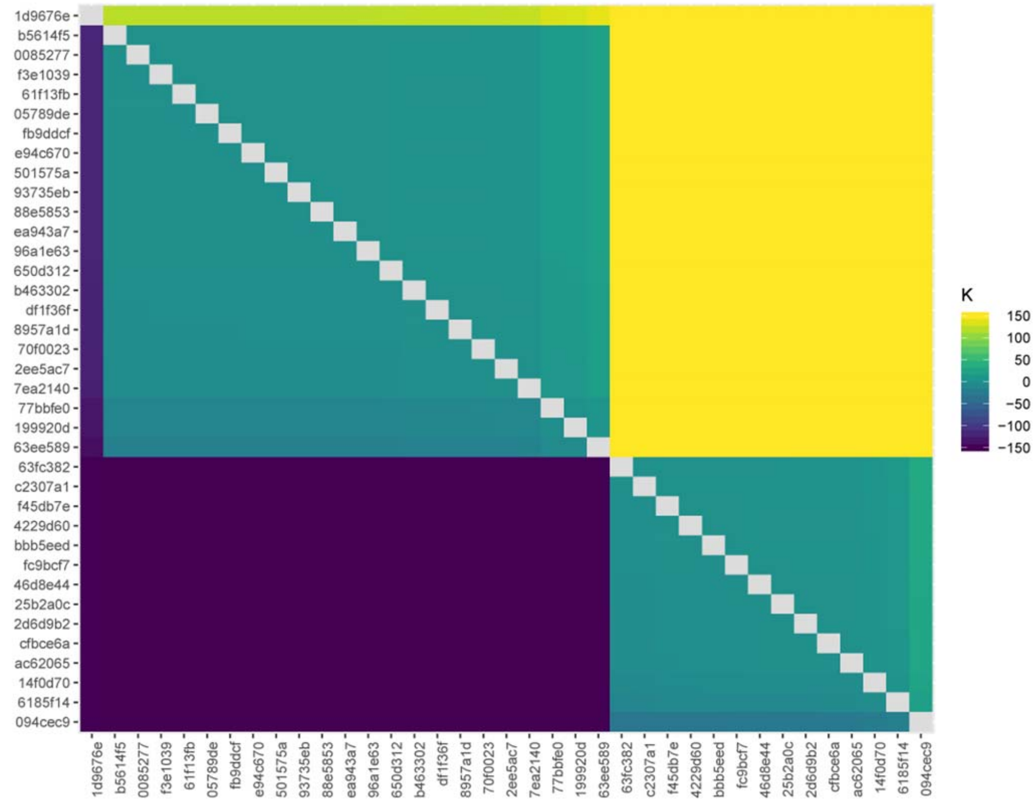

Supplementary Fig. 20

Heatmap showing the comparation of the Bayes factors for all model pairs. |K|>3 is considered as the significant threshold. Coordinates of the scale indicated the internal code for each model.

1d9676e

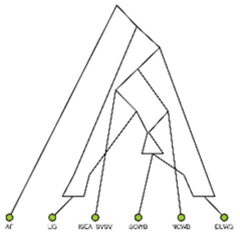

b5614f5

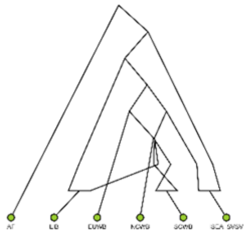

0085277

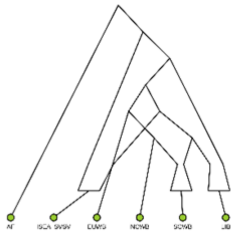

f3e1039

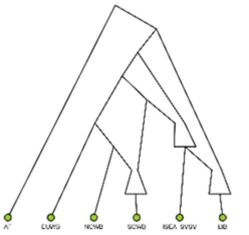

61f13fb

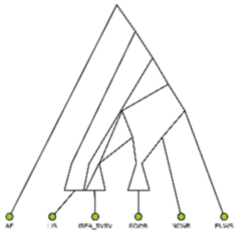

05789de

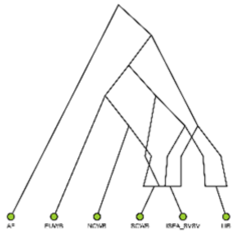

fb9ddcf

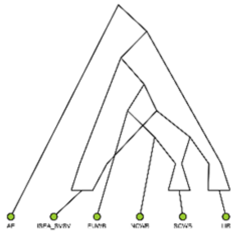

e94c670

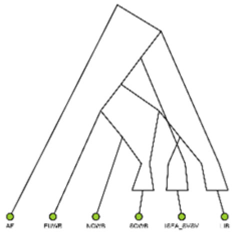

501575a

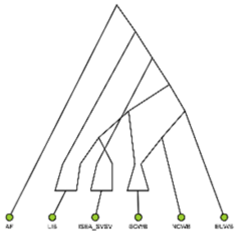

93735eb

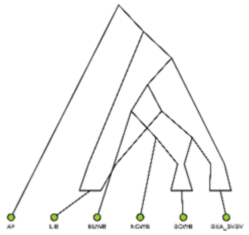

88e5853

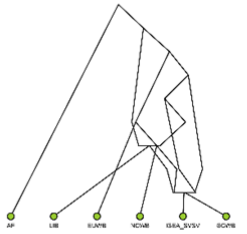

ea943a7

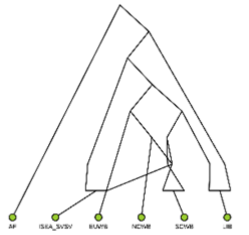

96a1e63

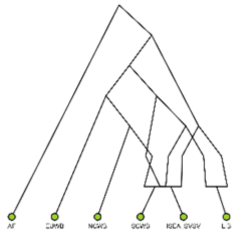

650d312

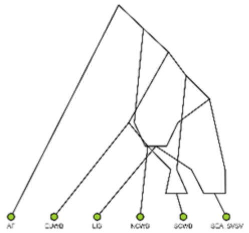

b463302

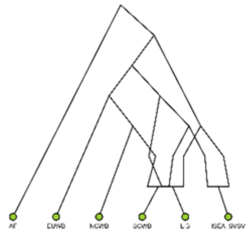

df1f36f

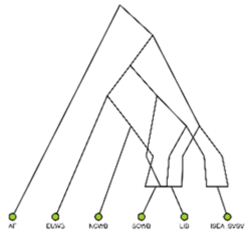

8957a1d

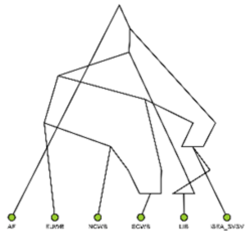

70f0023

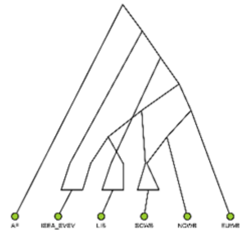

2ee5ac7

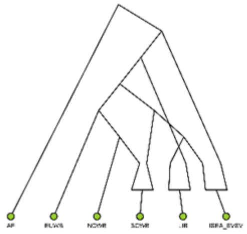

7ea2140

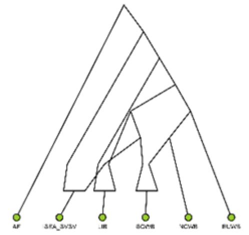

77bbfe0

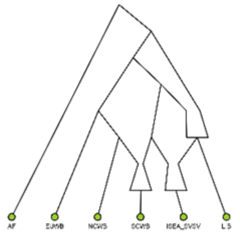

199920d

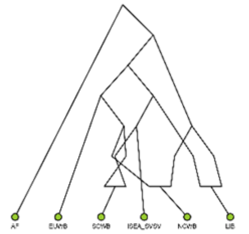

63ee589

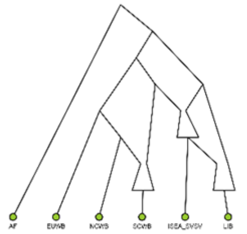

Supplementary Fig. 21

23 most well supported models (based on K) used for ADMIXTUREGRAPH. Strings on top of each diagram indicated the internal code for each model. (AF= *Phacochoerus africanus*; LIB=pygmy hog; ISEA_SVSV=*Sus verrucosus*; EUWB=European wildboar; NCWB=North Chinese wildboar; SCWB=South Chinese wildboar)

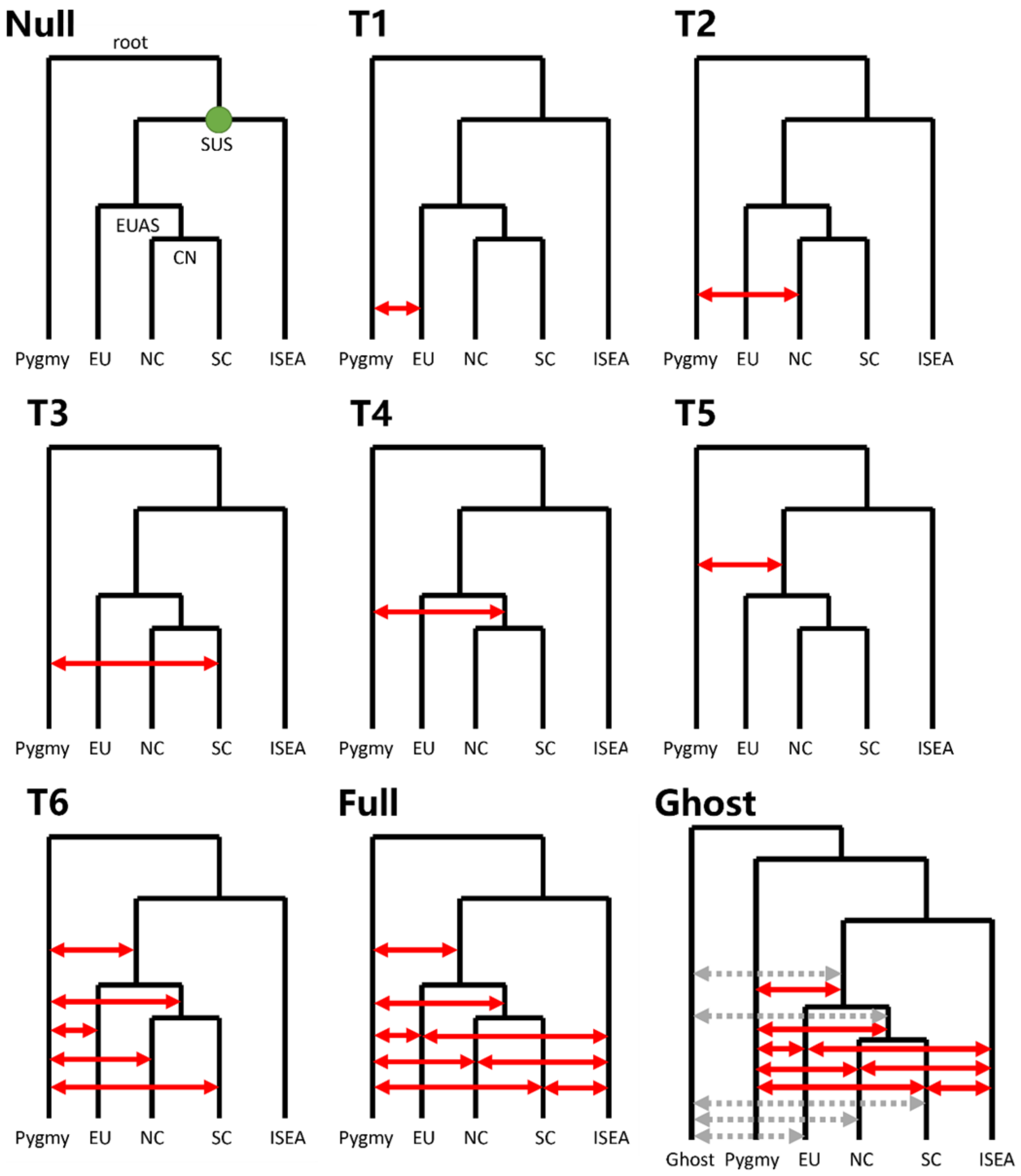

Supplementary Fig. 22

Demographic Models used for G-Phocs analyses. Two-way arrows indicated the migration band designated in each model. Gray dashed arrow indicated the migration band which ghost population involved. Green dot in Null model shows the fossil calibration. ISEA=Island of South Asia pigs, SC = South Chinese pig, NC = North Chinese pig, EU = European pig.

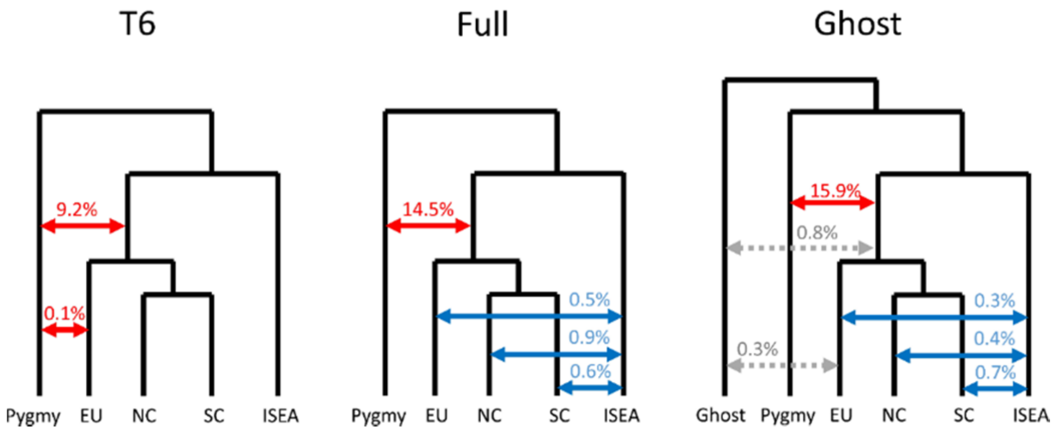

Supplementary Fig. 23

Migration bands are shown in blue, red and gray (see Supplementary Fig. 23) with associated values indicating estimates of total migration rates, which equal the probability that a lineage will migrate through the band during the time period when the two populations co-occur. Thig figure only show a subset of models tested and only significant migration signals were delineated (See Supplementary data 4 for further details). G-Phocs result is consistent with our admixture analysis between pygmy hog and *Sus scrofa*. In our model with ghost population, European pigs appeared to have more introgression from ghost population than other pigs, which supports the hypothesis that European pig used to hybrid with other species. Notably, G-Phocs always estimated a migration signal from Sus scrofa to other species. A possible reason is that Sus scrofa population remain the biggest effective population size and genetic diversity, which may overweight ILS. ISEA=Island of South Asia pigs, SC = South Chinese pig, NC = North Chinese pig, EU = European pig.

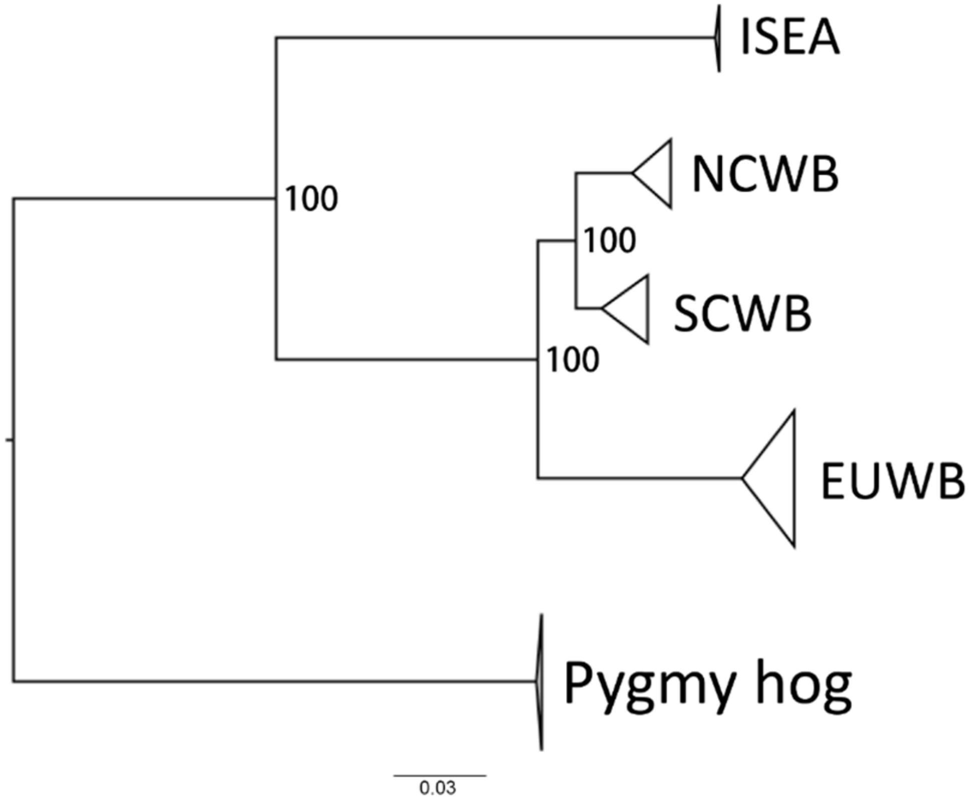

Supplementary Fig. 24

NJ tree based on selected 10,000 neutral loci used in G-PhoCS. Node labels show bootstrap values. The result is accordance with our main topology in consensus and concatenation tree. . ISEA=Island of South Asia pigs, SCWB = South Chinese wild boar, NCWB = North Chinese wild boar, EUWB = European wild boar.

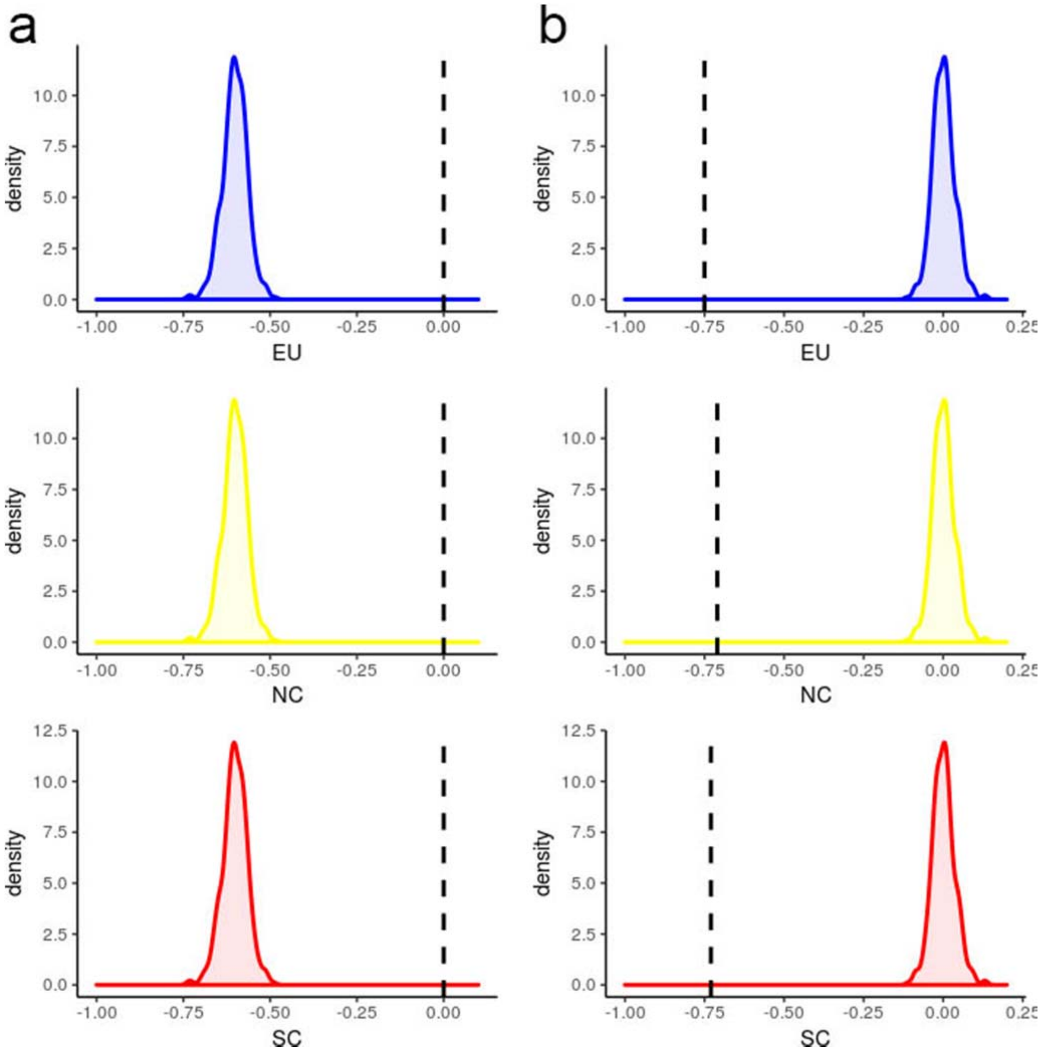

Supplementary Fig. 25

Null distributions of D. a) Each density curve corresponds to the D values (((ISEA, Pygmy hog), X), warthog) obtained under null models without gene flow, and the dash vertical line corresponds to the threshold value for the introgression fragments in the real data. b) Each density curve corresponds to the D values (((ISEA, X), pygmy hog), warthog) obtained under null models without gene flow, and the dash vertical line corresponds to the D values observed in the read data. All observed D values are significant ($P < 0.001$).

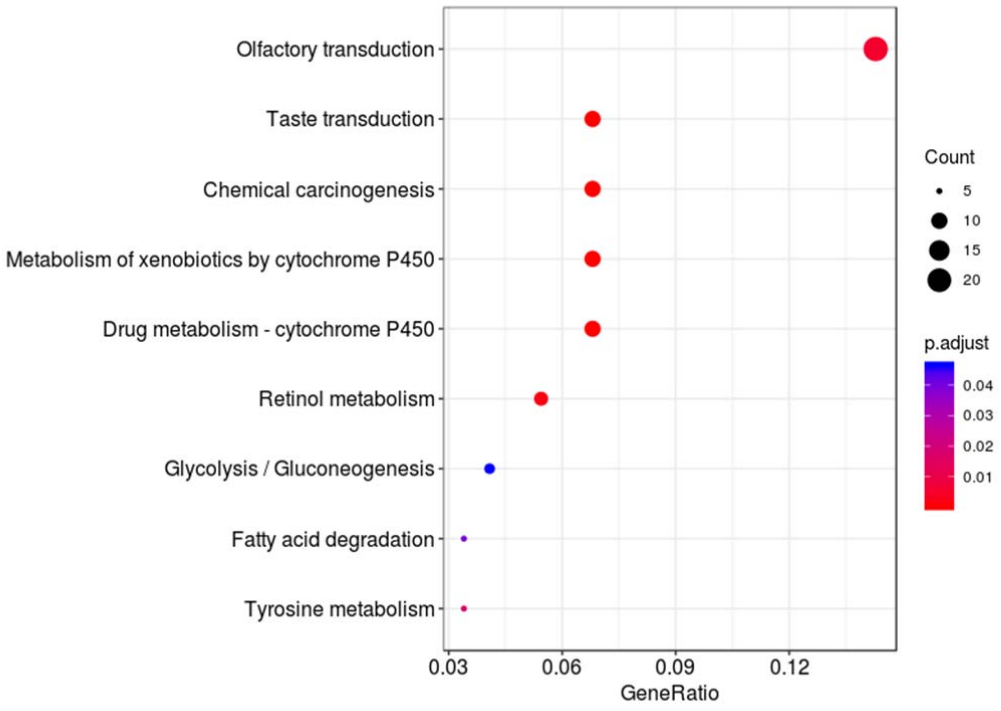

Supplementary Fig. 26

Functional enrichment result for pygmy hog/Sus scrofa introgression gene set

Analysis by gene ontology terms and pathways. The number of horizontal axis is the number of enriched genes. The bubble size indicates the ratio of genes in each term or pathway, and different colours correspond to different adjusted p-values. The p-values are adjusted by Benjamini-Hochberg method.

Supplementary Note

Phylogenomic analysis

We characterized the evolutionary history of pygmy hog by using one-to-one autosomal orthologous genes (1:1 - pig:cow). We applied both supermatrix and supertree phylogenomic methods based on maximum likelihood (ML) using *Babyrousa babyrussa* as an outgroup[3,4]. The concatenation and consensus methods resulted in same main topology (Fig. 2a, Supplementary Fig. 1, Supplementary Fig. 2). The phylogenetics analyses clearly show that *Suinae* consists of three separate clades, sub-Saharan suids, pygmy hog (*Porcula salvania*) and the genus *Sus*. The most basal split within the *Suinae* are sub-Saharan suids followed by a highly supported split of pygmy hogs (BS=100 in supermatrix and CF=1 in supertree) from all *Sus* species. There has been a longstanding taxonomic debate over the classification of pygmy hog as either basal to or nested inside the genus *Sus*[5,6]. Up to now, only one case of molecular classification has been reported for pygmy hog based on a short fragment of mitochondrial genome[2]. To compare our phylogenetics results to an earlier study using fragments of mitochondrial DNA, we carried out a Bayesian phylogenetic analysis using complete mitochondrial genomes (Supplementary Fig. 3). The resulting topology is consistent with previous studies confirming pygmy hog as basal to *Sus* and showing mitochondrial replacements between Sundaland Sus species[7].

Estimation of divergence time

We selected autosomal genomic loci supporting the main topology to obtain the basal divergence between the studied taxa (Fig. 2a, Supplementary Fig. 4). The divergence between *Babyrousa* and the other *Suidae* (sub-Saharan suids, pygmy hog and *Sus*) took place during middle Miocene ~14.2 Mya (95% HPD = 17.1-11.6). The basal split of *Suinae,* between extant sub-Saharan African Suidae and the Pygmy-Sus clade, took place during the late Miocene ~10.2 Mya (95% HPD = 12.7-7.9). This split was followed by the initiated diversification of the ancestors of extant sub-Saharan suids[8–11]. Our results show that the divergence between *Phacochoerus* and *Potamochoerus* during ~6.2 Mya (95% HPD = 7.7-4.5). Our divergence time estimates suggest that the initial divergence of the pygmy hog and *Sus* during Miocene/Pliocene boundary ~6.1Mya (95% HPD = 7.8-4.2). Within the Sus genus, DNA analysis suggests the two major groups of extant species (Sus scrofa and ISEA sus) diverged from a common ancestor in early Pliocene ~4.1Mya (95% HPD = 5.5-2.7).

Admixture between pygmy hogs and Sus

We used D-statistics (D) to test whether pygmy hogs had autosomal introgression from some present-day *Sus* species. We measured the difference in the percent matching by D (P1, P2, Pygmy hog, sub-Saharan suids) that does not differ significantly from zero when the derived alleles in the pygmy hog match alleles in the two Sus species/breeds equally often. If D is positive, pygmy hog alleles match alleles in the second Sus species (P2) more often, while if D is negative, pygmy hog alleles

match alleles in the first Sus species (P1) more often. We performed this test using 11 Sus populations (Supplementary data 2). We found that pygmy hogs have a clear overrepresentation of derived autosomal alleles with *Sus scrofa* compared to the Sus species from Island of south east Asian (ISEA) (Fig. 2b), suggesting that some admixture between pygmy hog and Sus scrofa may have occurred.

To further examine this autosomal genome-wide patterns of admixture between Pygmy hog and *Sus scrofa*, we calculated the D-statistics and fd-statistics in (ISEA, Pygmy hog, *Sus scrofa*, warthog) for every 100 kb non-overlapping window across the autosomal genome separately and used D and fd both >0 as a minimum threshold for introgressed intervals. In order to distinguish between introgression and ancestral variation, we also calculated DNA sequence divergence (dxy) for each candidate introgression interval and compared this with the autosomal chromosomal mean dxy, because introgressed regions generally show lower genetic divergence[12]. We compared the result between *Sus scrofa* from Europe, Northern China and Southern China. We identified 636 intervals of which 427 (67.1%) were shared among *Sus scrofa* (Supplementary Fig. 6), which suggested an ancestral gene flow between pygmy hog and the common ancestor of *Sus scrofa*.

X chromosome analysis

We reconstructed a phylogenetic tree based on the whole X chromosome and found remarkable inconsistency between the phylogenetic topology of the X chromosome and the autosomes, more specific in that pygmy hog significantly group with European and North Chinese *Sus scrofa* to the exclusion of Southern *Sus scrofa* and ISEA *Sus* species (Supplementary Fig. 8). To investigate this discrepancy, we used SAGUARO, which combines a hidden Markov model and a self-organizing map to infer both the trees and window boundaries. We mapped occurrences of the six most frequently observed unrooted ML topologies of six Suidae species along the 19 chromosomes (Fig. 3, Supplementary data 3, Supplementary Fig. 9). SAGUARO identified a ~40.6Mb (46.2-86.8 Mb) region in X chromosome, where pygmy hog clustered with ISEA and South China *Sus scrofa* breeds, while the European *Sus scrofa* clade appeared to be outgroup to this cluster. In the meantime, Northern Chinese domestic pig shows a splicing pattern, which clustered with European pig/North China pig in 46.2-57.1 Mbp and clustered with South China pig in 57.1-86.8 Mbp. To further confirm this observation, we carried out D-statistic analysis in sliding windows, expecting symmetry (D = 0) under the null hypothesis corresponding to no gene flow derived from the chromosomes (ISEA Sus, Sus scrofa, Pygmy hog, warthog) (Supplementary Fig. 10-15). Since the fixed frame-size sliding-window test may over-estimating the length of introgression region, for each quadruplet, we also ran individual SAGUARO analysis. The results suggest that within this genomic region, sequences of European/North China pigs have an ancient origin from a ghost lineage older that the split of pygmy hogs but younger than Sub-saharan suids. Due to the distinct haplotype between northern and southern Chinese *Sus scrofa* population in X chromosome, there are two prominent topologies draw in the putative introgression region. For those two genetic regions, we reconstructed phylogenetic tree separately

and obtained the same phylogenetic relationship among *Suinae* as SAGUARO estimated (Supplementary Fig. 16). We also identified the autosomal regions which support the introgression event found in the X chromosome. The sliding-window D-statistic analyses suggest a nearly equal amount of introgressed windows among different population (Supplementary Fig. 17). Similar average length of introgression region was estimated by the quadruplet SAGUARO analyses present (Supplementary Fig. 18).

D statistic under models of no gene flow

We used the divergence time estimated from MCMCtree. We assumed an effective population size of 100,000 individuals and a generation time of 5 years, a recombination rate 0.8 cM/Mb (0.8×10^{-8} per bp per generation) and a mutation rate 1.25×10^{-8}. All simulations were done in a msprime-based software momi2[13]. We simulated 10000 loci with length of 100kb:

```
#an simplify model for Suidae phylogeny without geneflow
model = momi.DemographicModel(N_e=1e5, gen_time=5,muts_per_gen=1.25e-8)
model.add_leaf("PYGMY", N=1e5)
model.add_leaf("EU", N=1e5)
model.add_leaf("NC", N=1e5)
model.add_leaf("SC", N=1e5)
model.add_leaf("ISEA", N=1e5)
model.add_leaf("AF", N=1e5)
model.move_lineages("NC","SC",t=800000)
model.move_lineages("EU","SC",t=900000)
model.move_lineages("SC","ISEA",t=4100000)
model.move_lineages("ISEA","PYGMY",t=6100000)
model.move_lineages("PYGMY","AF",t=10200000)
```

We compared these simulations with read data from our study. D-statstics were calculated using quadruplets (((ISEA, Pygmy hog), X), warthog) and (((ISEA, X), pygmy hog), warthog). Comparing the D values to the simulations under the null

model, we find that the observed D statistics are in the extreme tail, with $p < 0.001$ for all comparisons. Thus, these simulations suggest that it is very unlikely that D values that low are due to incomplete lineage sorting in the ancestral population, consistent with our claim of introgression. However, we need to be aware that this method requires a very detailed and precise demographic model to get a better assessment. The historical demographic information of pygmy hog and the ancestral population of Suinae species are still deficient. Here, we can only fit in a simplified model. Inaccurate of effective ancestral population size and bottleneck event may led to over-/underestimation of ILS.

Demographic model for Pygmy hog and *Sus*

To better assess the demographic implications of a separate species origin, rather than one due entirely to admixture, we performed demographic inference by applying G-PhoCS (Generalized Phylogenetic Coalescent Sampler) to simple branching models. G-PhoCS analyses are computational demanding, thus, due to computational constraints, we focused on 12 individuals, from the following different population or species: pygmy hog, ISEA (represented by *S. verrucosus* which has little detectable admixture with *Sus scrofa*), European wild boar, North and South China wild boar. Our objective was to infer rates of gene flow from between pygmy hog and *Sus scrofa* in the context of a complete demographic model that includes population divergence and changes in ancestral population sizes. We applied G-PhoCS to a multiple sequence alignment of neutral genome fragments of the 12 genomes. From this neutral dataset, we first constructed a NJ tree which is concordance with the main topology of the used population (Supplementary Fig. 24). We therefore assumed a plausible topology based on the main phylogenetic tree for the population phylogeny and fitted a variety of different gene flow models (Supplementary Fig. 22). G-PhoCS models' migration bands allow a test of admixture from D-statistic. Under the assumed branching structure, we inferred high probability of gene flow between the common ancestor of Sus scrofa and pygmy hog. Using G-PhoCS, we were also able to examine signatures of admixture in the ISEA population, and found significant gene flow between the ISEA *Sus* and Asian wild boar, which is consisted with former research[7]. Furthermore, we also applied a model with a basal ghost population to test the results obtained from the X-chromosome analysis. This model confirmed a post-speciation gene flow between the common ancestor of wild boar and the ghost population (Supplementary data 4, Supplementary Fig. 23). Also, European wild boar shows a secondary gene flow from the ghost population. We interpret this pattern as isolation by distance, as mainland Asia is the start point of *Sus scrofa* expansion, which allow the ancestor of extant Asian pig have more chance to hybrid with non-introgression population. As we can't properly match a non-introgression population into our model, G-PhoCS may assign the exceeding introgression allele in European wild boar as a potential gene flow signal.

Enrichment analysis

We applied a functional annotation analysis using PANTHER v.11[14] on the candidate introgressed genes (Supplementary data 5). Genes from the pygmy hog/Sus scrofa introgression and the Sus scrofa/ghost lineage introgression were analyzed separately. Gene-enrichment analyses were performed using clusterProfiler[15]. False discovery rate (FDR) was performed to adjust P-values using the Benjamini and Hochberg method. A P-value of <0.05 was used as the cut-off criterion. For the pygmy hog/Sus scrofa introgression gene set, significant enriched KEGG pathway was showed in Supplementary Fig. 27. For the Sus scrofa/ghost lineage introgression, we only identified a limited number of candidate genes we identified, and no significant enrichment was found.

References

1. Bosse, M. *et al.* Genomic analysis reveals selection for Asian genes in European pigs following human-mediated introgression. *Nat. Commun.* **5**, 4392 (2014).
2. Funk, S. M. *et al.* The pygmy hog is a unique genus: 19th century taxonomists got it right first time round. *Mol. Phylogenet. Evol.* **45**, 427–436 (2007).
3. Stamatakis, A. RAxML version 8: A tool for phylogenetic analysis and post-analysis of large phylogenies. *Bioinformatics* **30**, 1312–1313 (2014).
4. Frantz, L. A. F. Speciation and domestication in Suiformes: a genomic perspective. (Wageningen University, 2015).
5. Hodgson, B. H. On a new form of the hog kind or Suidae. *J. Asiat. Soc. Bengal 16,* **16**, 423–428 (1847).
6. Oliver, W. L. R. & Deb Roy, S. The pygmy hog (Sus salvanius). *Pigs, Peccaries Hippos status Surv. Conserv. action plan. IUCN, Gland* 121–129 (1993).
7. Frantz, L. A. F. *et al.* Genome sequencing reveals fine scale diversification and reticulation history during speciation in Sus. *Genome Biol.* **14**, R107 (2013).
8. Gongora, J. *et al.* Rethinking the evolution of extant sub-Saharan African suids (Suidae, Artiodactyla). *Zool. Scr.* **40**, 327–335 (2011).
9. Brunet, M. & White, T. D. Deux nouvelles espèces de Suini (Mammalia, Suidae) du continent Africain (Éthiopie; Tchad). *Comptes Rendus l'Academie Sci. - Ser. IIa Sci. la Terre des Planetes* **332**, 51–57 (2001).
10. Haile-Selassie, Y. & Simpson, S. W. A new species of Kolpochoerus (Mammalia: Suidae) from the Pliocene of Central Afar, Ethiopia: Its Taxonomy and Phylogenetic Relationships. *J. Mamm. Evol.* **20**, 115–127 (2013).
11. Pickford, M. Ancestors of broom's pigs. *Trans. R. Soc. South Africa* **67**, 17–35 (2012).
12. Smith, J. & Kronforst, M. R. Do Heliconius butterfly species exchange mimicry alleles? *Biol. Lett.* **9**, 20130503–20130503 (2013).
13. Tortereau, F. *et al.* A high density recombination map of the pig reveals a correlation between sex-specific recombination and GC content. *BMC Genomics* **13**, 586 (2012).
14. Westbury, M. V *et al.* Extended and Continuous Decline in Effective Population Size Results in Low Genomic Diversity in the World's Rarest Hyena Species, the Brown Hyena. *Mol. Biol. Evol.* **35**, 1225–1237 (2018).
15. Yu, G., Wang, L.-G., Han, Y. & He, Q.-Y. clusterProfiler: an R Package for Comparing Biological Themes Among Gene Clusters. *Omi. A J. Integr. Biol.* **16**, 284–287 (2012).
