# Supplementary material for: Genomic analysis on pygmy hog reveals extensive interbreeding during wild boar expansion
Source: Nat Commun. 2019 Apr 30;10:1992. doi: 10.1038/s41467-019-10017-2 (PMC6491599; doi:10.1038/s41467-019-10017-2)
Supplement: Supplementary file 3 — Description of Additional Supplementary Files [file 41467_2019_10017_MOESM3_ESM.pdf]

Description of Additional Supplementary Files

File Name: Supplementary Data 1
Description: Sample information, sequencing and mapping statistics.

File Name: Supplementary Data 2
Description: Results from whole genome D-statistics analysis.

File Name: Supplementary Data 3
Description: Numbers of chromosomal segments and sites, and median length of segments supporting alternative rooted topologies identified by saguaro.

File Name: Supplementary Data 4
Description: Main set of parameter estimates in the G-PhoCS analysis.

File Name: Supplementary Data 5
Description: Genes with introgression signal and its corresponding functional pathway category.
